# INTRAGRO: A machine learning approach to predict future growth of trees under climate change

**DOI:** 10.1002/ece3.10626

**Published:** 2023-10-20

**Authors:** Sugam Aryal, Jussi Grießinger, Nita Dyola, Narayan Prasad Gaire, Tribikram Bhattarai, Achim Bräuning

**Affiliations:** ^1^ Institut für Geographie Friedrich‐Alexander‐Universität Erlangen‐Nürnberg Erlangen Bayern Germany; ^2^ Institute of Tibetan Plateau Research Chinese Academy of Sciences, State Key Laboratory of Tibetan Plateau Earth System, Resources and Environment (TPESRE) Beijing China; ^3^ Laboratoire sur les écosystèmes terrestres boréaux, Département des Sciences Fondamentales Universitédu Québec à Chicoutimi Chicoutimi Quebec Canada; ^4^ Department of Environmental Science, Patan Multiple Campus Tribhuvan University Lalitpur Nepal; ^5^ Central Department of Biotechnology Tribhuvan University Kathmandu Nepal

**Keywords:** dendrometer, growing‐season change, intra‐annual growth, machine learning algorithm, Pinus roxburghii, sub‐tropical Nepal

## Abstract

The escalating impact of climate change on global terrestrial ecosystems demands a robust prediction of the trees' growth patterns and physiological adaptation for sustainable forestry and successful conservation efforts. Understanding these dynamics at an intra‐annual resolution can offer deeper insights into tree responses under various future climate scenarios. However, the existing approaches to infer cambial or leaf phenological change are mainly focused on certain climatic zones (such as higher latitudes) or species with foliage discolouration during the fall season. In this study, we demonstrated a novel approach (INTRAGRO) to combine intra‐annual circumference records generated by dendrometers coupled to the output of climate models to predict future tree growth at intra‐annual resolution using a series of supervised and unsupervised machine learning algorithms. INTRAGRO performed well using our dataset, that is dendrometer data of *P. roxburghii* Sarg. from the subtropical mid‐elevation belt of Nepal, with robust test statistics. Our growth prediction shows enhanced tree growth at our study site for the middle and end of the 21st century. This result is remarkable since the predicted growing season by INTRAGRO is expected to shorten due to changes in seasonal precipitation. INTRAGRO's key advantage is the opportunity to analyse changes in trees' intra‐annual growth dynamics on a global scale, regardless of the investigated tree species, regional climate and geographical conditions. Such information is important to assess tree species' growth performance and physiological adaptation to growing season change under different climate scenarios.

## Graphical abstract

This study presents a novel approach called INTRAGRO, which combines dendrometer data and climate model outputs to predict future growth at an intra‐annual resolution. This approach has the advantage of being applicable to any tree species, climate or geographical condition and can provide valuable information on the change in cambial activity on a global scale.

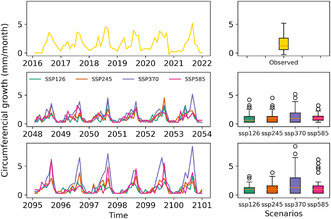

## 1 INTRODUCTION

Climate change imposes severe effects on global ecosystems in many aspects. Some impacts of climate change on terrestrial ecosystems are indicated by changes in ecosystem structure, species range shifts and phenology due to warming and increased frequency, severity and duration of extreme events (Pörtner et al., 2022). Changes in plant phenology can be observed worldwide and are mainly attributed to shifts in climate seasonality, especially caused by changes in temperature (Piao et al., 2015; Zhang et al., 2022) and precipitation (Wu et al., 2022). The phenological trends, however, reveal a strong dependency on regional or local changes in temperature, photoperiod (day length), nutrients and water availability (Piao et al., 2019). This geographical dependency of the growing season indicates the necessity of more regional studies and methodological developments worldwide, especially in the tropical and subtropical regions of Asia, Africa and South America, where little information on current and future plant phenology is available (Pörtner et al., 2022). Most existing studies used remote‐sensing data to study the beginning and termination of the growing season. Remote sensing evidently provides important and practical tools in broad‐scale vegetation studies (Babst et al., 2019; Shen et al., 2021). However, the major drawback of using remote‐sensing data is the so‐called ‘scale issue’ (Wu & Li, 2009). Remote sensing cannot provide the option to analyse a specific species and might not be helpful in specific regions like very cloudy areas or some tropical evergreen forests, in which trees do not discolour or shed their leaves (Rathgeber et al., 2022). Besides, the leaf phenology does not account for the seasonal variation in radial growth (cambial phenology) of trees, because leaf and cambial phenology do not necessarily coincide (Gričar et al., 2022). In addition, changes in leaf phenology may not adequately reflect the abrupt cessation of cambial activity caused by drought during the growing season or growth re‐stimulation initiated by favourable climate conditions (Fajstavr et al., 2019).

Mechanistic growth models mainly use a small number of input data to predict the intra‐annual growth (cambial phenology) in species or at the individual tree level. The Vaganov–Shashkin (VS) model is one of the frequently used mechanistic models in dendrochronological studies to infer the intra‐annual cell formation of trees using inter‐annual tree‐ring width (Vaganov et al., 2006, 2011). The VS model simulates tree‐ring width based on daily variations in environmental conditions, such as temperature and soil water availability, allowing researchers to understand the impact of short‐term climatic events on tree growth, making it a powerful tool for studying both past and current climatic conditions. Previous studies have successfully applied the VS model in high‐elevation (He et al., 2017; Yang et al., 2017) and high‐latitude environments (Anchukaitis et al., 2006; Tumajer, Buras, et al., 2021; Tumajer, Kašpar, et al., 2021; Wu et al., 2020), for which the model was actually designed. However, its application in tropical and subtropical climates is still questionable. Like other mechanistic models, it needs a parametrisation of several endogenous and exogenous parameters, making its application critical at places with limited information about different environmental and edaphic factors. Buttò et al. (2020) reported a substantial decrease in model performance while applying it in a wet site, reflecting the limitation of the model in some specific environmental conditions. Another limitation of the VS model is the requirement of annual tree‐ring series of a species, which can be a critical factor in its application in tropical areas, where the majority of species do not form detectable growth rings (Alves & Angyalossy‐Alfonso, 2000; Quesada‐Román et al., 2022). The mentioned limitations of existing approaches demand the creation of a technique that can be applied in high spatiotemporal resolution, regardless of geographical and climate conditions. Dendrometers are electronic devices widely used to record tree stem diameter or circumference variations with high temporal resolution. They provide the opportunity to record the growth change in trees at individual and stand levels, also under tropical climate conditions (Butz et al., 2017; Spannl et al., 2016).

Depending on specific research objectives, dendrometers can record data in intervals ranging from minutes to weeks. Most studies have used dendrometers to investigate the current growth status of trees and their response to short‐term weather changes in various climate conditions (Biondi & Hartsough, 2010; Deslauriers et al., 2007; Drew et al., 2008; Fan et al., 2019; Häusser et al., 2021; Raffelsbauer et al., 2019; van der Maaten et al., 2013). Recently, there have been technical developments in data acquisition and analytical approaches (Aryal, Häusser, et al., 2020; Deslauriers et al., 2011; van der Maaten et al., 2016), providing techniques to relate the sub‐daily to the yearly growth patterns of trees to the local climate. When implementing the output of regional and/or global climate models (GCMs), the observed relationships can be extrapolated to model the trees' intra‐annual growth under future climate conditions. The new generation of climate models (CMs) provides an enormous opportunity for an improved prediction of future climate scenarios (Li et al., 2021). The high spatial and temporal output of CMs has already been used in several sectors for planning and policymaking and in forest management by accessing the impact of global warming and its residual effects (Del Martinez Castillo et al., 2022; Wang, Liu, et al., 2022; Wang, Waters, et al., 2022). The current study aims to introduce a novel approach, ‘Inter‐ and Intra‐annual Growth Predictor’ (here and after known as ‘INTRAGRO’), which analyses and models trees' intra‐ and inter‐annual growth under current and future climate conditions, irrespective of the climatic seasonality or the local site conditions. In this approach, we combined dendrometer recordings and the model output from CMs under different climate change scenarios.

## 2 METHODS

### 2.1 Study area and species description

The study site was selected to analyse the seasonal growth variation of Chir pine (*Pinus roxburghii*) trees, with a special focus on the growth dynamics during the early growing season. *P. roxburghii* is an extremely important timber species in Nepal, and better knowledge of its growth dynamics may improve management plans in pine plantations under climate change conditions. The study site represents an even‐aged pine plantation with an open canopy structure covering about three hectares on the campus of Tribhuvan University, Kathmandu, Nepal, at an elevation of 1320 m asl. The analysed trees were selected based on their stem shape (straight, upright without sign of wounding or irregular growth) and full access to sunlight.

**FIGURE 1:**
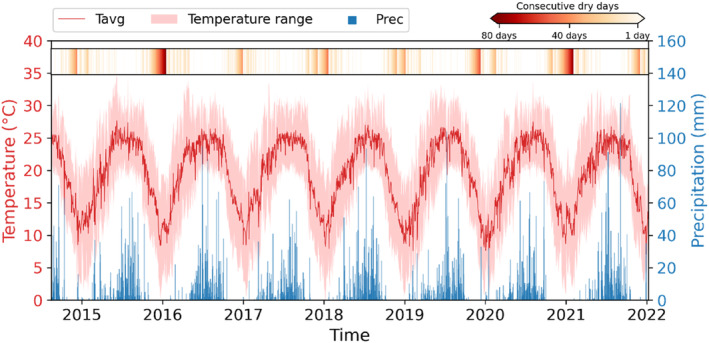
Daily resolved temperature and precipitation of the study area along with the temporal distribution and duration of periods with consecutive dry days. Prec, daily precipitation; Tavg, daily average temperature.

For subsequent climate analyses, we used the daily resolved climate data from Tribhuvan International Airport (TIA) in Katmandu (http://www.mfd.gov.np/city?id=31), which is located nine kilometres east of the study site at an elevation of 1350 m asl. The study area experiences a subtropical climate characterised by an unimodal temperature and precipitation pattern (Figure 1). During the wet summer monsoon season (June–September), 78% of the annual precipitation amount is received, accompanied by a high mean seasonal (June–September) temperature of 24.52°C. The early spring to spring or pre‐monsoon season (mid‐February to May) is generally considered as the dry period of the year (15% of total annual rainfall), with dry soil moisture conditions and rapidly warming temperatures from 13.43°C in winter (December to mid‐February) to 20.29°C. The study site experiences extended drier periods during the winter and pre‐monsoon seasons, with the longest dry period observed for 81 days, lasting from 30 October 2015 to 18 January 2016. However, the dry period in the pre‐monsoon season is more pronounced due to higher evapotranspiration as a result of higher temperatures compared with the winter.

### 2.2 Analytical steps of INTRAGRO approach

**FIGURE 2:**
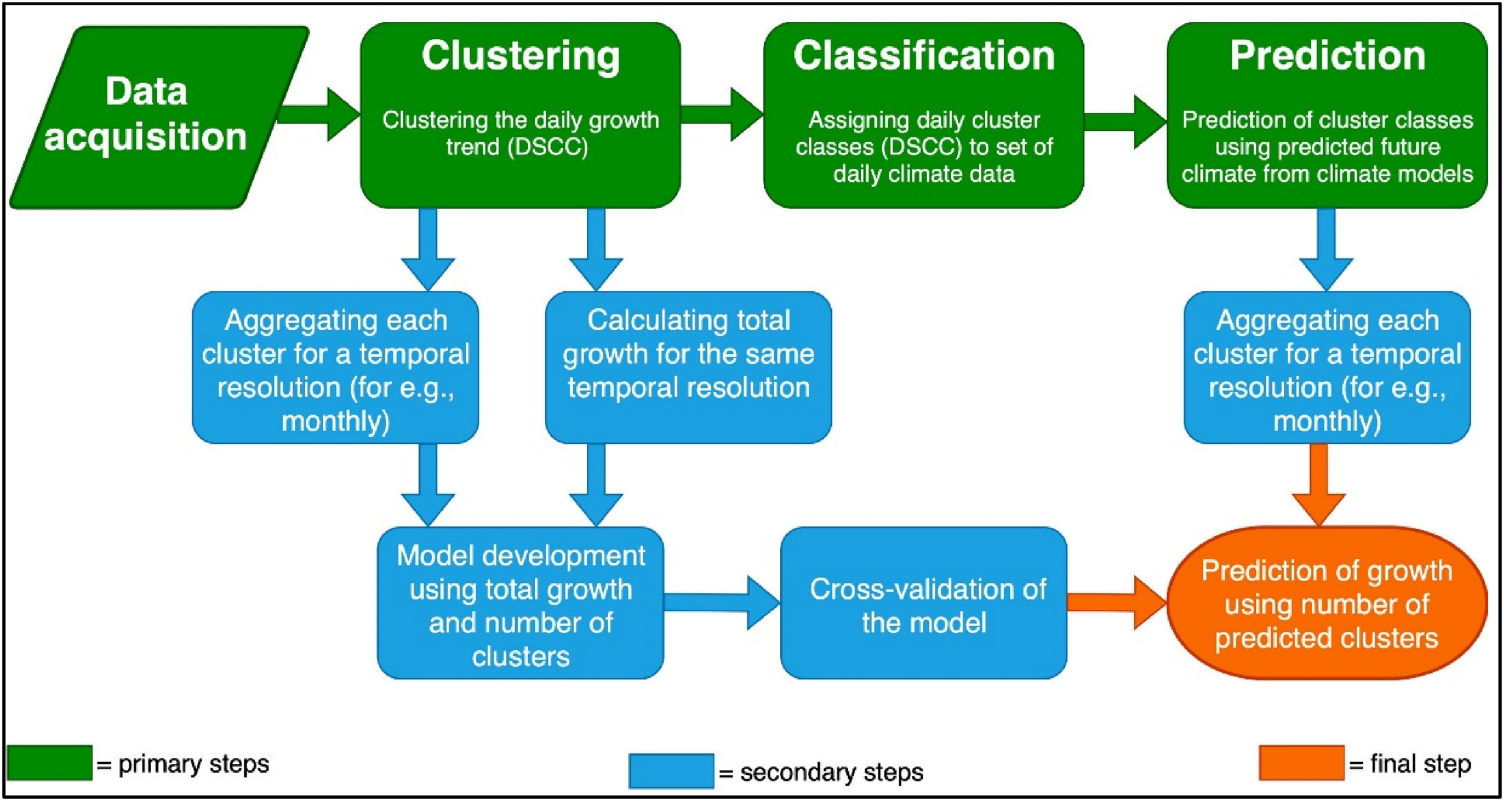
Overall workflow of the INTRAGRO approach. CMs, climate models.

The analytical INTRAGRO approach broadly consists of four individual analytical steps of data acquisition, clustering, classification and prediction (Figure 2). The individual steps in the workflow are elaborated in detail below.

#### 2.2.1 Growth data acquisition and pre‐processing

**FIGURE 3:**
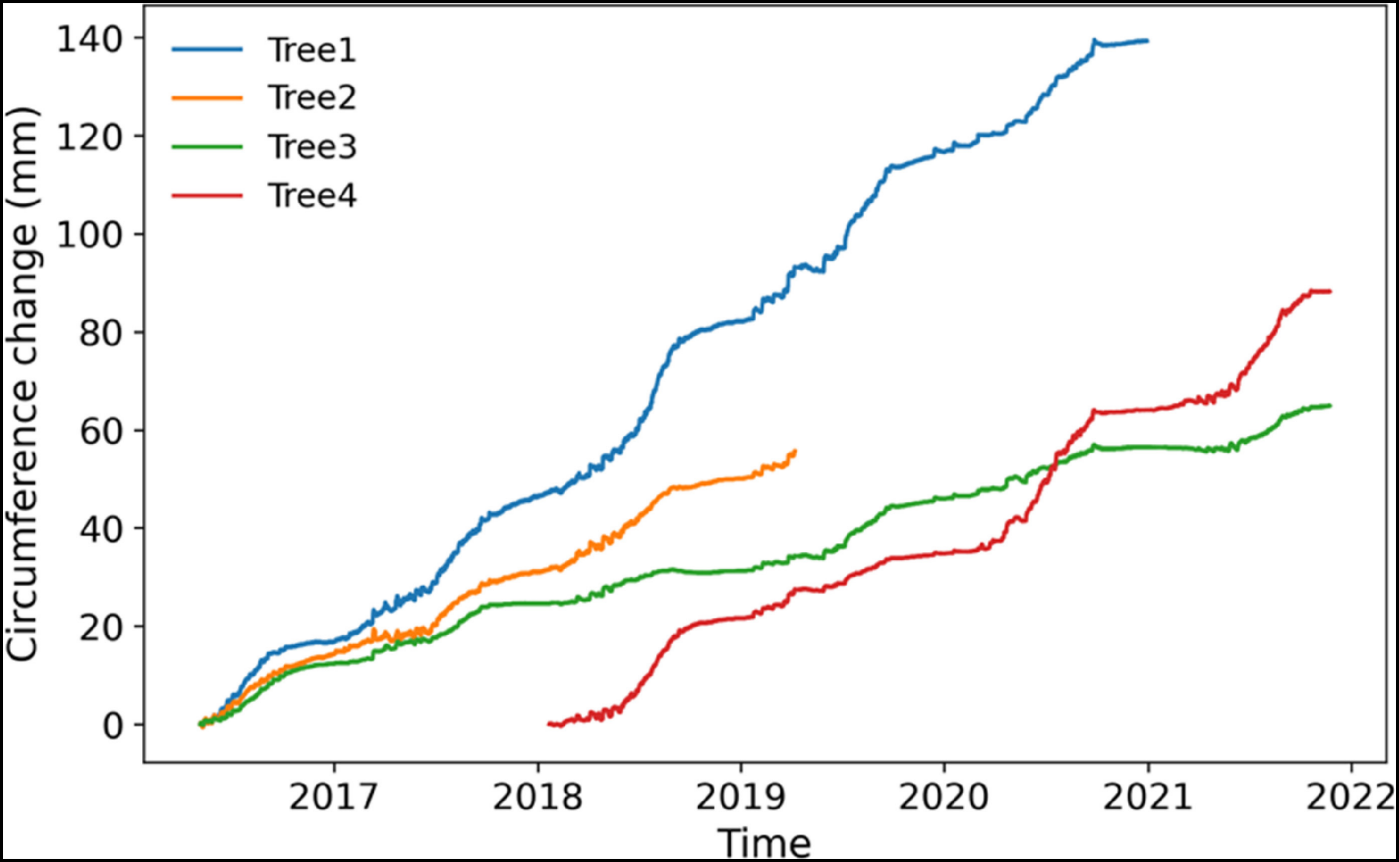
Dendrometer time series of the four studied trees from April 2016 to October 2021.

We installed electronic band dendrometers (DRL26, EMS Brno) on four pine trees of similar size. To avoid a strong impact of swelling and shrinking of the bark on the dendrometer data, we removed the outer bark layers without wounding the cambial zone. We collected dendrometer data in a 30‐min resolution spanning 5 years from 2016 to 2021. Three dendrometers (T1, T2 and T3) started recording in April 2016, whereas due to technical errors, dendrometer T4 started its measurement in January 2018 (Figure 3). The final measurement dates for T1 and T2 were December 2020 and March 2019, respectively, whereas dendrometers T3 and T4 included measurements until October 2021. Altogether, the measurement period covered 2020 days from 5 May to 2016 to 22 November 2021. Data gaps and jumps in the dendrometer data were removed using the R‐package ‘dendRoAnalyst’ (Aryal, Häusser, et al., 2020). We used the ‘spline.interpolation’ function to fill data gaps shorter than 24 h, whereas gaps persisting for more than 24 h were interpolated by applying the ‘network.interpolation’ function using the other dendrometer time series. Furthermore, the jumps (sudden positive or negative change in recording) in the dataset due to the regular adjustment of the dendrometer band because of stem growth or because of battery replacement were removed using the function ‘jump.locator’. Afterwards, we calculated the daily circumference change (DCC) as a difference between the circumferences at consecutive morning maxima.

#### 2.2.2 Clustering

The course of daily stem circumference variation patterns (DSCCs) shows typical cycles, with stem shrinkage during daytime, when trees transpire more water than is replenished by the root system, and stem increment during nighttime due to stem water replenishment and irreversible growth caused by cambial activity (Deslauriers et al., 2007; Zweifel, 2016). Besides the leaf phenological status of the tree, its DSCCs depend on the short‐term weather conditions, showing characteristic diurnal patterns of stem increment and shrinkage, with variable amplitudes of DCC and radial growth during different seasons, but also during short‐term climatic episodes within one season (Raffelsbauer et al., 2019). The advantage of clustering DSCC patterns is that patterns occurring during different seasons of the year but representing similar plant physiological responses to the weather conditions are merged in one group of data, whereas in conventional time series analyses, tree responses are interpreted within the seasonal climate pattern (e.g. Spannl et al., 2016). This also provides opportunities to analyse the tree physiological responses in climates showing weak or no clear climate seasonality. Clustering also allows us to use shorter, non‐continuous dendrometer series due to technical problems or short measurement periods, which are usually rejected despite their information content.

The final processed dataset was rearranged in a way that all data for 1 day stand in one row. To avoid statistical overfitting (López, 2022), we introduced noise in the dataset by stacking the individual days from different trees in one dataset. We used an unsupervised machine learning technique to cluster the DSCCs. For time series clustering, we applied the ‘tslearn’ (Tavenard et al., 2020) module, a Python package that provides tools to apply relevant models to time series data. In particular, the ‘TimeSeriesKMeans’ function was used with dynamic time warping (DTW) as metrics. Dynamic time warping can compare time series of variable size and is robust to shifts (in the tempo of record) across the time dimension (Cuturi & Blondel, 2017). The Kruskal–Wallis test (Kruskal & Wallis, 1952), followed by Dunn's post‐hock test (Dunn, 1961), was applied to identify the daily climate conditions occurring in each cluster. Before clustering, we first tested different cluster numbers using the Elbow method (Thorndike, 1953) to obtain the optimal number of clusters.

#### 2.2.3 Classification of climate variables

The cluster classes of daily SCV patterns (DSCCs) were subsequently used to train a supervised classification of the climate data. This step used the daily resolved climate data of minimum, maximum temperature and precipitation from Kathmandu Airport (http://www.mfd.gov.np/city?id=31). Furthermore, the daily minimum and maximum temperatures were used to calculate potential evapotranspiration (PET) using Hargreave–Samani (HS) formula (Hargreaves & Samani, 1985) and then the daily moisture index (MI) by subtracting daily PET from daily precipitation. For the classification, we applied the random forest (RF) technique in the Scikit‐learn module (Pedregosa et al., 2011). The RF classifier uses a combination of tree classifiers. Each classifier is generated by selecting a random vector sampled independently from the input vector, and each tree casts a unit vote for the most popular class to classify an input vector (for more details, see Breiman (2001)). We used daily minimum and maximum temperature (Tmin, Tmax) and daily precipitation (Prec) as predictors and cluster classes (DSCC) as the target variables. Since the occurrence of the clusters showed strong seasonality, ‘month’ was also included as a predictor for better model performance. We used 60% of the data (predictor and target variable) as a training dataset, and the remaining 40% were used to validate the model performance. To further validate our final model performance, we used the F1‐score introduced by (van Rijsbergen, 1979) (Equation 1), where the F1‐score of one represents perfect model performance.

Equation 1: Formula to calculate the F1‐score of the classification model.

(1) $$F1\;\text{score}=\frac{\mathrm{tp}}{\mathrm{tp}+\frac{1}{2}\left(\mathrm{fp}+\mathrm{fn}\right)}$$

where tp = true‐positive classification, fp = false‐positive classification and fn = false‐negative classification by the model.

#### 2.2.4 Prediction of future cluster classes using the output of GCMs

##### Evaluation of global climate models and statistical downscaling

Monthly data of temperature and precipitation of several global climate models (GCMs) were obtained from KNMI Climate Explorer (https://climexp.knmi.nl/selectfield_cmip6.cgi?id). To assess the best CGM for our dendrometer‐climate evaluation, we computed the Kling–Gupta efficiency (KGE) score (Gupta et al., 2009) (Equation 2) using the monthly observed climate data from Kathmandu (Table S1). We selected the INM‐CM5‐0 dataset from the Institute of Numerical Mathematics (INM) (Volodin et al., 2019) based on its comparatively higher KGE score for all climate variables. We evaluated 31 GCMs for temperatures and 39 GCMs for precipitation to get the best representative GCM for the study area. The INM‐CM5‐0 dataset scored 0.80 and 0.67 KGE values for minimum and maximum temperature and 0.79 for precipitation. Since a KGE value of more than 0.5 is considered a good model performance (Knoben et al., 2019), INM‐CM5‐0 was the best option among all considered CMs. After selecting the best‐performing GCM in our study area, we used the model's simulated daily climate data and Kathmandu's observed daily climate data to downscale the model data. To downscale the temperature data, we employed the simple RF regression technique (Jose et al., 2022). The simulated and observed daily temperatures were regressed for the observed period. The trained model was then used to predict the future climate in different scenarios. In contrast, we used the so‐called two‐step RF technique to downscale precipitation (Chen et al., 2021). In this method of precipitation downscaling, the RF classification method was first used to classify days with and without rainfall events. In the second step, rainfall amounts for each event were calculated using the RF regression technique, as similarly performed with the temperature data. We repeated the same downscaling methods on four Shared Socio‐economic Pathways (SSPs): SSP126, SSP245, SSP370 and SSP585 of the Sixth version of the Coupled Model Intercomparison Project (CMIP6). The downscaling resulted in a daily series of corrected temperature and precipitation data until 2100.

Equation 2. Formula to calculate KGE between instrumental and climate data of climate models (CMs).

(2) $$\text{Kling}–\text{Gupta efficiency}\;\left(\mathrm{KGE}\right)=1-\sqrt{{\left(r-1\right)}^{2}+{\left(\beta -1\right)}^{2}+{\left(\gamma -1\right)}^{2}}$$

where *r* = Correlation; $\beta =\frac{\mu_{s}}{\mu_{o}}$; γ=σsμsσoμo; *μ*_s_ = mean of simulated climate; *μ*_o_ = mean of observed climate; *σ*_s_ = standard deviation of simulated climate; *σ*_o_ = standard deviation of observed climate.

##### Prediction of the cluster classes for future GCM scenarios

We subsequently used the already trained RF classification model from section 2.2.3 to further predict daily clusters (DSCC) using daily downscaled climate (output of Evaluation of global climate models and statistical downscaling) for various scenarios. The model used provided daily climate variables to assign a cluster class for each corresponding day until the end of 2100. We defined two 6‐year periods in this study to reduce the computational expense. We chose the periods 2048–2053 (mid‐21st century) and 2095–2100 (late‐21st century) and subsequently termed/named them as ‘2050s’ and ‘2100s’ in our subsequent analyses.

#### 2.2.5 Modelling growth and number of clusters

To model the monthly radial tree growth, we aggregated the number of each cluster (DSCC) in monthly resolution into the two observation periods, ‘2050s’ and ‘2100s’. A linear model was formed using monthly aggregated circumference change (GRO) and the number of each cluster class for the observation period and cross‐validated using the ‘K‐fold’ validation approach (Hastie et al., 2001), dividing the whole calibration period into 10 consecutive time blocks. Among 10‐fold, nine consecutive time blocks were selected for the model calibration, and the remaining one was used for verification, yielding verification statistics for each iteration. Hence, we obtained 10 values for the coefficient of determination (*R* ^*2*^), variance explained (*VE*) and root mean square error (*RMSE*). The cross‐validation was performed using the Scikit‐learn module (Pedregosa et al., 2011). We repeated the calibration and verification procedure six times. The first four times were performed for each cluster class, then an additional two for a group of clusters, clusters showing positive increment and vice versa. Finally, the monthly aggregated number of clusters in the two selected periods, the 2050s and 2100s, were used as predictors in the validated model to predict the monthly circumference change (GRO). We used cluster numbers instead of climate as a proxy for growth prediction because the monthly aggregated cluster numbers showed a more robust relationship with tree growth than climate variables (Figure S1). Another fact that encourages us to use cluster numbers is the inconsistent temporal relationship of growth with a climatic variable over a complete growing season (Dobbert et al., 2021; Häusser et al., 2021), since unstable growth–climate relationships can result in comparatively less precise predictions. We, therefore, additionally used generalised additive models (GAM) (Hastie & Tibshirani, 1986) to model the daily cumulative circumference change in each year for observed and predicted periods using the ‘mgcv’ package (Wood, 2006) and used its first derivative to calculate the daily growth rate in R software (R Development Core Team, 2021). The modelled cumulative circumference change for each period was used to derive the statistical onset and cessation of the growing season, as explained by Fan et al. (2019) and Malik et al. (2020). In particular, we consider the 95% limit of cumulative growth as a threshold such that the days of the year when cumulative growth passed 2.5% and 97.5% of total value were considered onset and cessation dates, respectively.

## 3 RESULTS

### 3.1 Clustering of daily growth patterns

**FIGURE 4:**
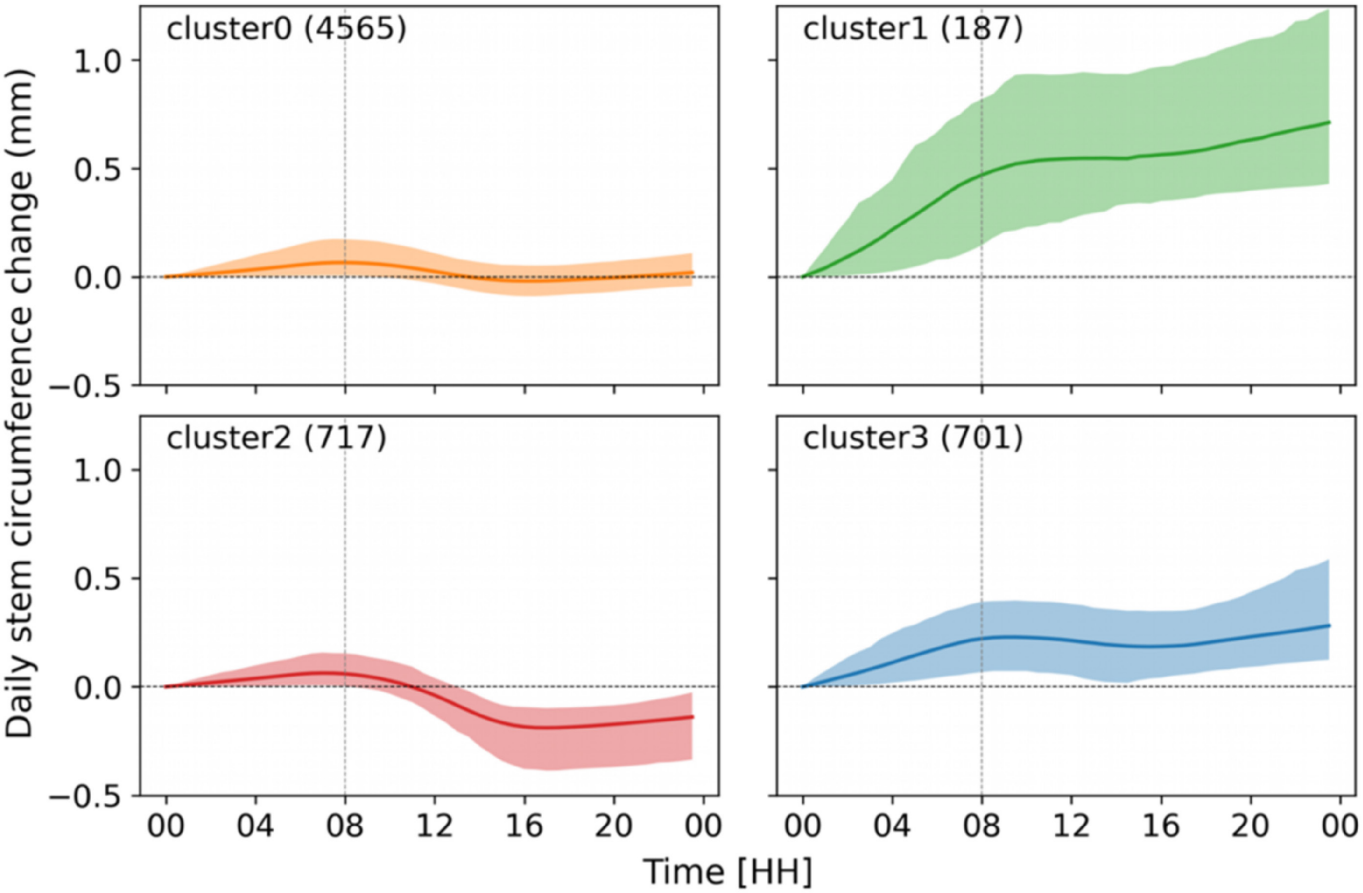
Pattern of daily stem circumference variations in mm for the four clusters: cluster0, cluster1, cluster2 and cluster3. The numbers in brackets represent the total number of daily cycles. HH, hours.

The test for the optimum cluster number based on the Elbow method revealed four clusters in the dataset (Figure S2). Our dendrometer data showed four distinct DSCC patterns, namely cluster0, cluster1, cluster2 and cluster3 (Figure 4). All DSCCs include a general trend of daily radial change, which can be described as a rapid circumference increase until 8 a.m. Depending on the cluster class, the stem circumference either increases or decreases afterwards. Among the four clusters, cluster1 and cluster3 showed a positive daily net change in stem circumference. Therefore, together, they form the ‘growing cluster group’ (GC). On the contrary, cluster0 and cluster2 represent a stable or negative daily net change in stem circumference. These clusters were, therefore, combined into the ‘non‐growing group’ (NGC).

**FIGURE 5:**
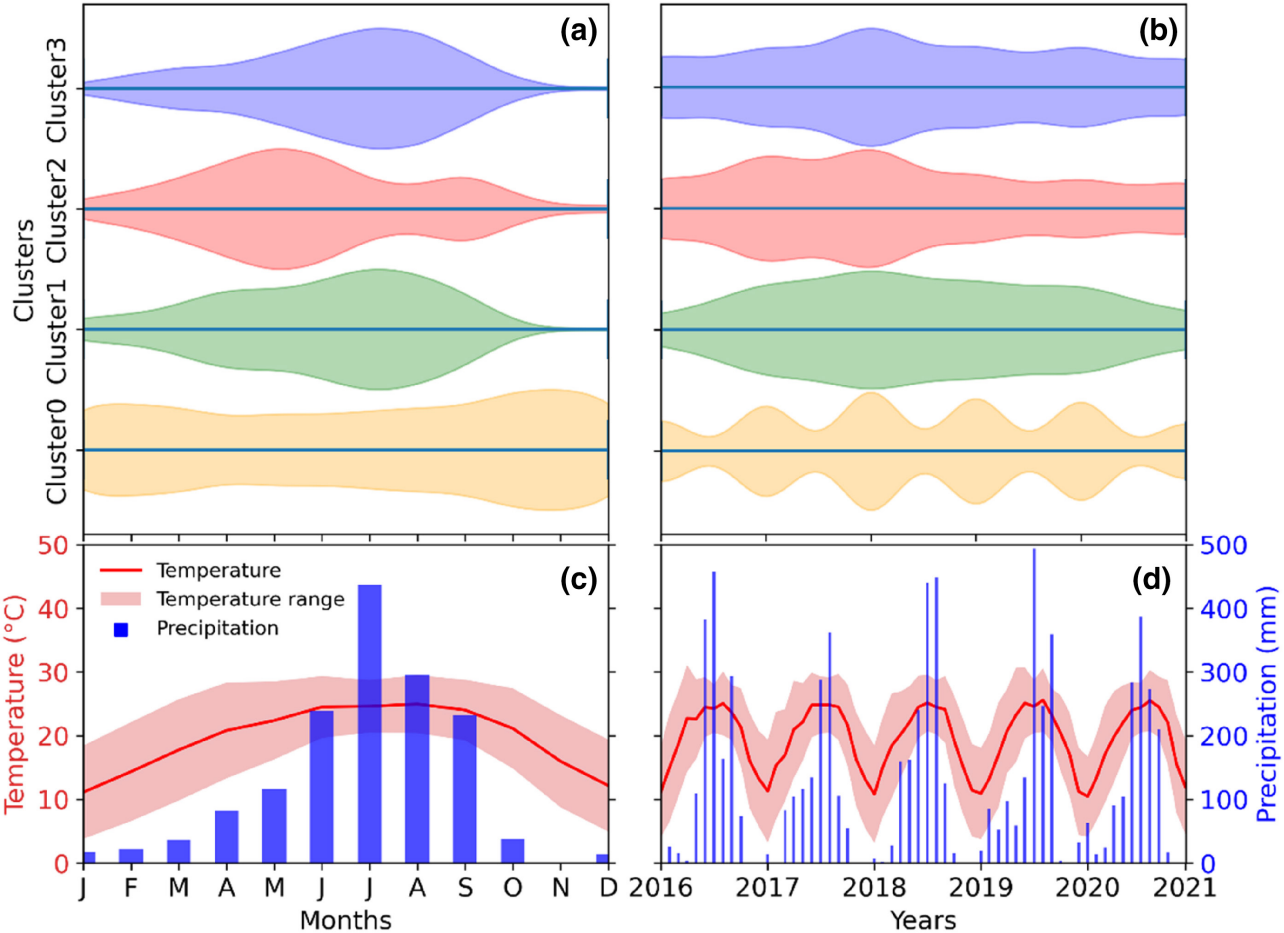
Intra‐annual (a) and inter‐annual (b) distribution of stem diameter variation clusters in the observed period. The lower panel represents the monthly temperature and precipitation averaged for 2016–2020 (c) and the annual pattern of monthly temperature and precipitation for each year from 2016 to 2020 (d).

The most frequent cluster (cluster0) was observed during the winter season, with a total occurrence of 74% (Figure 4). The occurrence of the least frequent cluster1 (3% of the total clusters) was concentrated in the rainy summer monsoon season, when trees do not experience moisture stress. Cluster3, with a frequency of 11%, has two peaks of occurrence (Figure 5a). The first peak occurs during the pre‐monsoon season (especially in April), while the second peak occurs during the summer monsoon season (especially in July and August). Cluster2, with 12% of occurrence, is concentrated in the pre‐monsoon season, a warm and dry period at our study site (Figure 5c). The yearly distribution of the clusters revealed that all growth patterns, except cluster0, increased in frequency until 2018 and then decreased afterwards (Figure 5b). In contrast, cluster0 showed wiggly, more or less regular occurrence throughout the study period.

### 3.2 Classification of daily climate with the observed daily growth pattern

We identified climate conditions for each DSCC by employing a supervised classification of the predefined clusters with the corresponding daily climate (minimum, maximum temperature and total precipitation) condition. The RF classification performed well in both the training and test datasets, with 84% and 74% accuracy, respectively (Table 1).

**TABLE 1:** Summary table showing the statistics of the model's performance during classification for the training and test datasets.

Model's performance in the training dataset (60%)				
Accuracy of the model: 0.84				
	Precision	Recall	F1‐score	Support
Cluster0	0.83	0.98	0.90	2354
Cluster1	0.88	0.55	0.68	181
Cluster2	0.92	0.59	0.72	636
Cluster3	0.80	0.64	0.71	531
Accuracy			0.84	3702
Macro avg	0.86	0.69	0.75	3702
Weighted avg	0.85	0.84	0.83	3702

Model's performance in the test dataset (40%)				
Accuracy of the model: 0.74				
	Precision	Recall	F1‐score	Support
Cluster0	0.80	0.88	0.83	1766
Cluster1	0.59	0.50	0.54	87
Cluster2	0.56	0.43	0.48	348
Cluster3	0.54	0.43	0.48	267
Accuracy			0.74	2468
Macro avg	0.62	0.56	0.58	2468
Weighted avg	0.72	0.74	0.73	2468

*Note*: Accuracy: the ratio of the number of correct predictions to the total number of input samples. Precision: the ratio of the number of correct positive predictions to the number of all positive predictions (including incorrect positive predictions). Recall: the ratio of the number of positive predictions to the number of actual positives in observed data. Macro avg: the average F1‐score without considering the proportion of each cluster in the dataset. Weighted avg: the weighted average F1‐score with the proportion of each cluster in the dataset. Support: the number of each cluster in the dataset.

**FIGURE 6:**
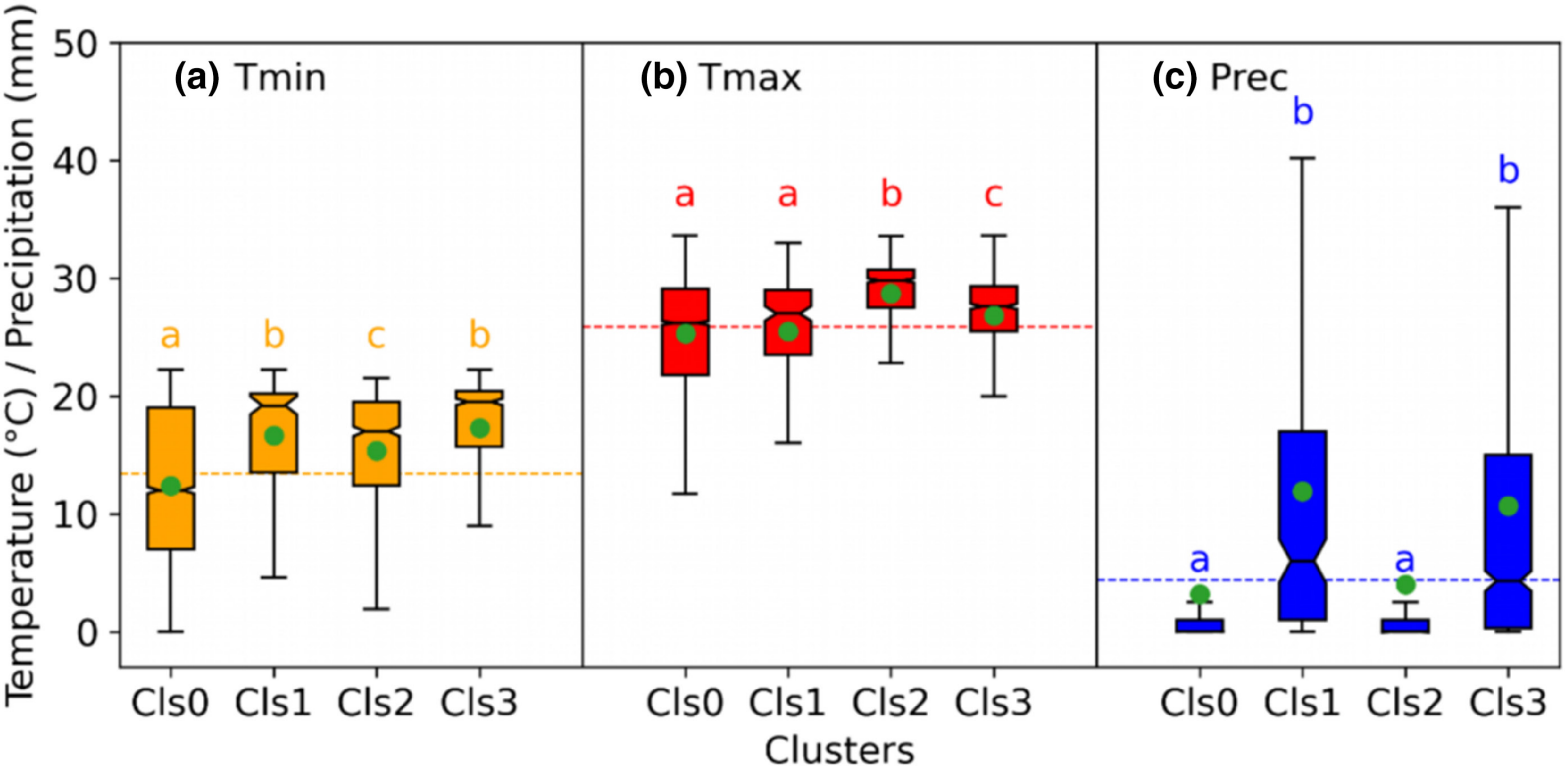
Temperature (a, b) and precipitation (c) conditions for the four clusters 0–3 of stem circumference variations. Different letters in the clusters represent significant differences at the *p* < .05 level based on the Kruskal‐Wallis test followed by Dunn's post hoc test analysis. The horizontal lines represent the overall mean, and the green points in each cluster represent the average climate of each cluster. Cls0 = cluster0; Cls1 = cluster1; Cls2 = cluster2; Cls3 = cluster3; Tmin = Minimum temperature; Tmax = Maximum temperature; and Prec = Precipitation.

From the classification, we identified the complex interaction between temperature and precipitation, resulting in the occurrence of four weather patterns, in the way that DSCC cluster0 mainly occurs in cold, dry weather, whereas cluster2 occurs during warm, dry conditions (Figure 6). Cluster1 and cluster3 occur during days with precipitation but significantly differ in maximum temperature. Cluster3 occurs when the maximum temperature is significantly higher than in cluster1. Cluster0 occurs during colder conditions when maximum and minimum temperatures are substantially lower than in cluster2. Thus, cluster0 is mainly concentrated on colder days with average minimum and maximum temperatures of 12.4 ± 6.3 and 25.3 ± 4.4°C (Figures 5 and 6). Precipitation amount, corresponding to soil moisture availability, is the primary weather factor separating the growing and non‐growing cluster groups.

### 3.3 Change in daily growth patterns under climate change scenarios

**FIGURE 7:**
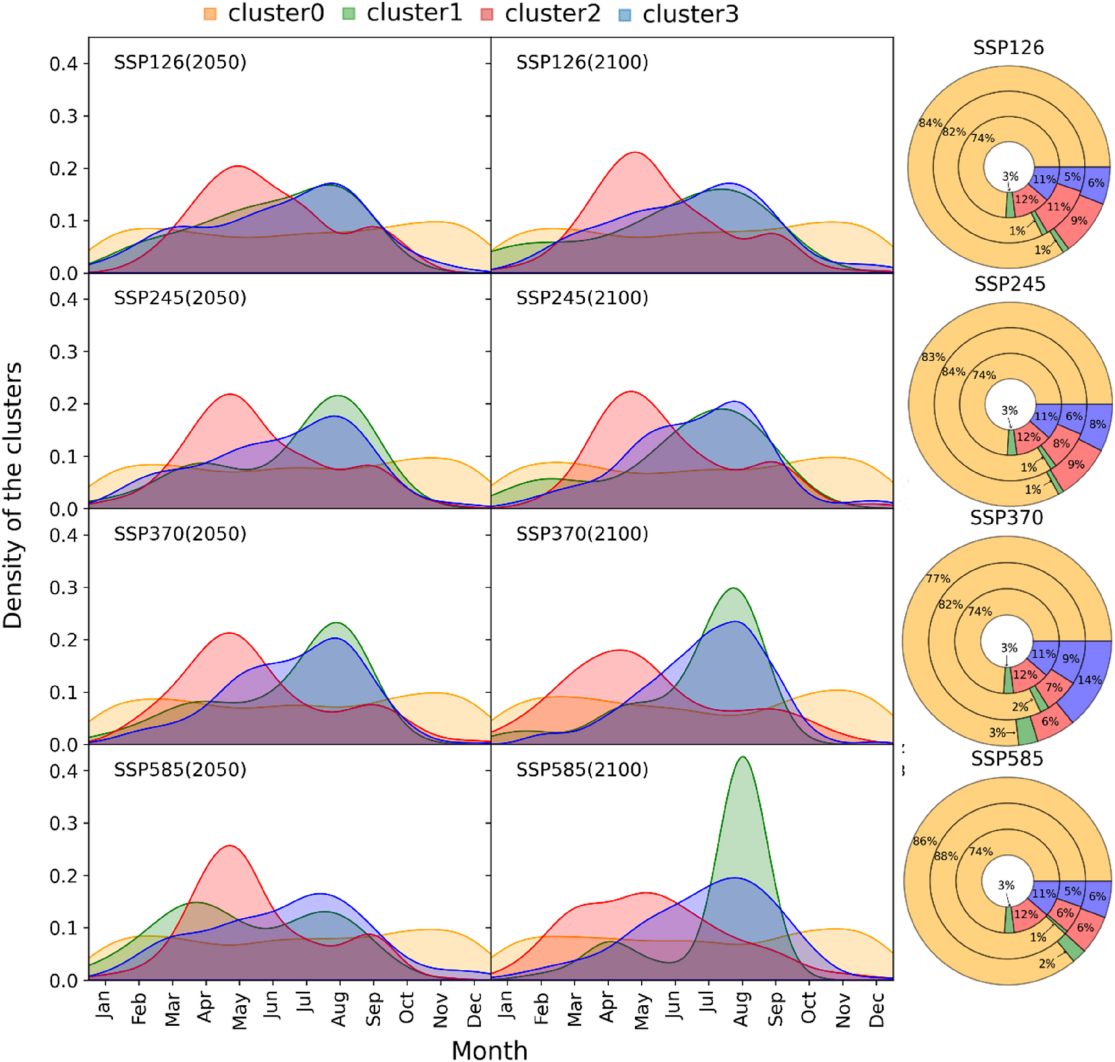
Modelled distribution of stem circumference variation clusters (DSCCs) under different climate change scenarios. The graphs in columns A and B represent the predicted distribution of DSCCs in the 2050s and 2100s. Column C represents the proportions of DSCCs 0–3 in different climate change scenarios. In the concentric diagram, the inner, middle and outer circular layers represent the observed period (2016–2021) and the predicted periods 2048–2053 and 2095–2100 for different Shared Socio‐economic Pathways (SSPs).

For predicting the future occurrence of daily DSCCs, we used the daily maximum and minimum temperature and precipitation from the INM‐CM5‐0 dataset on the trained model. Significant changes in the distribution of the modelled DSCC clusters in future scenarios are apparent (Figure 7). The forecast shows an increased occurrence of cluster0. However, the proportion of change varies between the different climate change scenarios. Except for the SSP375 in both study periods, the predicted proportion of cluster0 increases in all months. The cluster0 frequency will decrease during the core monsoon (July, August and September) in both study periods for SSP375. In contrast, the other clusters will decrease in number during the 2050s and slightly increase during the 2100s in all scenarios. Cluster1 is predicted to decrease in all scenarios during the 2050s and further increase in the 2100s, especially in SSP126 and SSP245. However, its observed temporal distribution (January to September) will not change. In all scenarios, cluster1 will be more concentrated around August, with a greater increase in SSP370 and SSP585 during the 2100s. The intra‐annual distribution pattern of cluster2 shows two peaks during the drier climate conditions in the pre‐monsoon and post‐monsoon seasons. The proportion of cluster3 will also decrease under future climate conditions except for the scenarios SSP245 and SSP374. In SSPs 245 and 370, the proportion of cluster3 will significantly increase during the monsoon season (June–September).

### 3.4 Relationships between the number of clusters and radial tree growth

**FIGURE 8:**
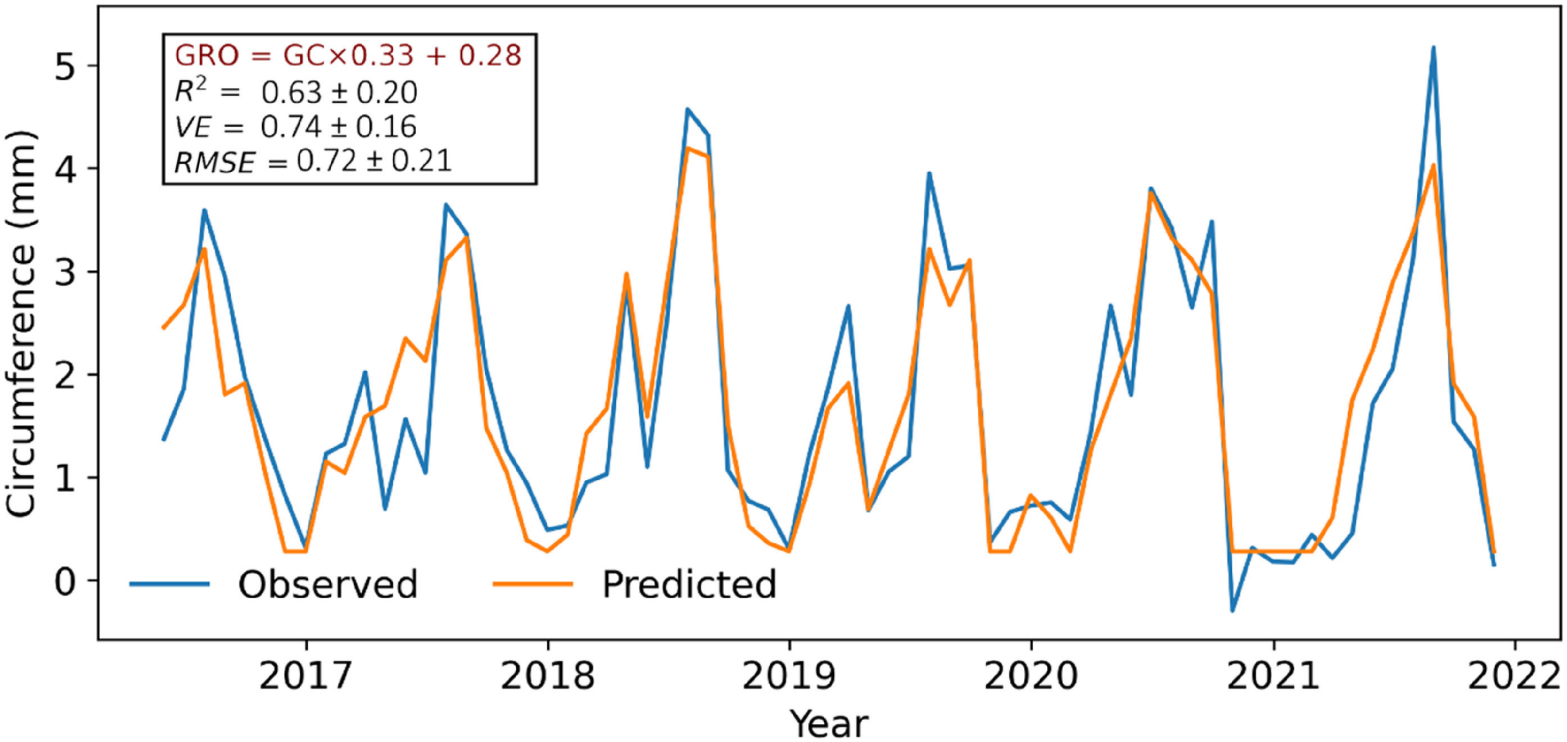
Comparison of observed and simulated monthly circumferences.

The monthly aggregated counts of DSCC clusters showed strong associations with total monthly growth (GRO). The correlation with the monthly aggregated cluster0 and cluster2 were −0.59 (*p* < .01) and 0.17 (*p* < .05), respectively, whereas the two growing groups (GCs) cluster1 and cluster3 showed more robust relationships of 0.76 (*p* < .01) and 0.81 (*p* < .01) (Figure S1). The relationship became even stronger when the number of clusters in the GC and the NGC were combined: the combined GC clusters showed a stronger relationship with the aggregated growth (0.89, *p* < .01) than the NGC (−0.51, *p* < .01). Due to this strong relationship, we calculated a linear model using monthly aggregated growth and monthly aggregated number of DSCC clusters in the GC (Figure S3). The 10‐fold cross‐validation further corroborates the robustness and stability of the model with an average coefficient of the determinant (*R* ^*2*^) value of 0.63 ± 0.20 (*p* < .01), explained variance (*VE*) of 0.74 ± 0.16 and a minimal root mean square error (*RMSE*) of 0.72 ± 0.21 (Figure 8). The linear model simulated the monthly growth very close to the observations, further highlighting the model's overall robustness.

**FIGURE 9:**
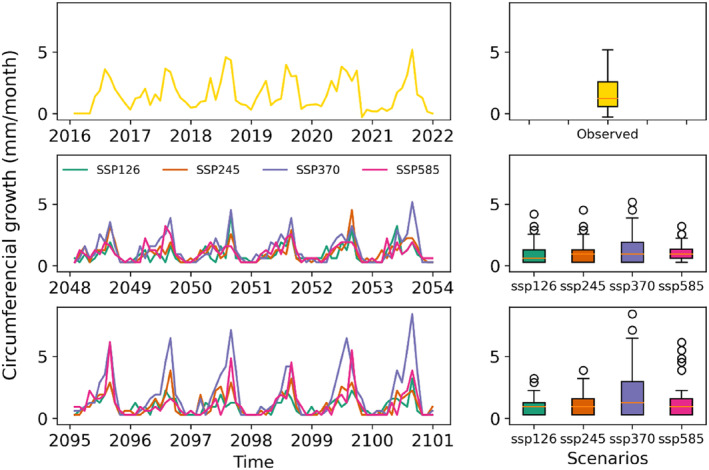
Predicted tree growth in the 2050s and 2100s under different climate change scenarios.

Accordingly, we used the linear model to predict the monthly growth in the two predefined periods, 2050 and 2100, using the monthly aggregated numbers of clusters belonging to the growing phase as a predictor. The resulting modelled growth followed the same general intra‐annual patterns as in the observation period, however with a reduced growth at the beginning of the growing season, a maximum growth during the summer monsoon season and a subsequent gradually decreasing growth in the post‐monsoon season (Figure 9). The median growth amount is not significantly different in all three sub‐periods except for the SSP370. SSP370 shows enhanced growth in both predicted periods, reaching its highest values in the 2100s.

A deeper analysis of the intra‐annual growth patterns reveals that compared with observations, tree growth will drop during January–March in all scenarios during both periods. The main growing season period from April to October shows scenario‐specific growth changes in the future. In SSP126, trees will likely experience more than 40% growth reduction in both periods, with the highest reduction during the summer monsoon season (Table 2). However, the trees will increase growth by 10% in early winter (December) during the 2100s. The growth pattern in SSP245 will be similar to SSP126, but with less intense reduction. The trees will experience 36.7% and 32.5% growth loss during the 2050s and 2100s, respectively. However, in addition to December, trees will also show positive growth in May during the 2100s. Interestingly, in SSP370, most of the months during the summer monsoon season are likely to experience a significant growth increase during the 2100s, first leading to slightly suppressed growth by 17.8% during the 2050s, which will turn to growth enhancement by 20.4% during the 2100s. Finally, in the case of SSP585, tree growth will decrease by 35.6% during the 2050s despite expected enhanced growth in May and December. Similarly, trees will reduce growth by 28.6% during the 2100s.

**TABLE 2:** Percentage change in monthly growth in different scenarios in the 2050s and 2100s and change in starting of the growing season (SOS), end of the growing season (EOS), and total growing period length (GS) in days with the positive and negative value showing earlier and later than observed period.

Periods	Scenarios	Growth change (%) compared with observed values for corresponding months													Δ days		
		Jan	Feb	Mar	Apr	May	Jun	Jul	Aug	Sep	Oct	Nov	Dec	Total	SOS	EOS	GS
2050s	SSP126	−36.5	−36.1	−18.2	−51.6	−23.6	−31.7	−73.5	−29.9	−67.5	−37.2	−34.9	−30.7	−44.0	−5	8	13
	SSP245	−50.5	−20.2	−33.0	−29.4	−8.4	−37.0	−63.3	−19.3	−45.2	−57.9	−34.9	−17.1	−36.7	2	4	2
	SSP370	−50.5	−36.1	−44.1	−18.3	**33.4**	**4.9**	−44.3	**12.7**	−27.8	−23.4	−53.3	−17.1	−17.8	13	−16	−29
	SSP585	−50.5	−36.1	−3.4	−25.7	**6.8**	−50.1	−45.7	−43.6	−50.1	−44.1	−34.9	**23.6**	−35.6	6	19	13
2100s	SSP126	−43.5	−41.3	−33.0	−40.5	−0.8	−42.2	−57.4	−40.6	−47.6	−57.9	−34.9	**10.0**	−40.9	2	22	20
	SSP245	−57.5	−36.1	−51.5	−51.6	**18.2**	−5.6	−51.6	−16.2	−47.6	−57.9	−44.1	**23.6**	−32.5	8	14	6
	SSP370	−43.5	−25.5	−58.9	**0.1**	**63.8**	**46.7**	**27.4**	**70.4**	**21.8**	−16.5	−53.3	−3.6	**20.4**	24	−29	−53
	SSP585	−43.5	−51.9	−58.9	−33.1	−12.2	−34.4	−57.4	**23.3**	−32.7	−9.6	−53.3	−17.1	−28.6	10	−7	−17

*Note*: The reddish colored cells indicate negative change, while the greenish colored cells with bold values signify positive change.

### 3.5 Comparison of observed and modelled intra‐annual growth patterns

**FIGURE 10:**
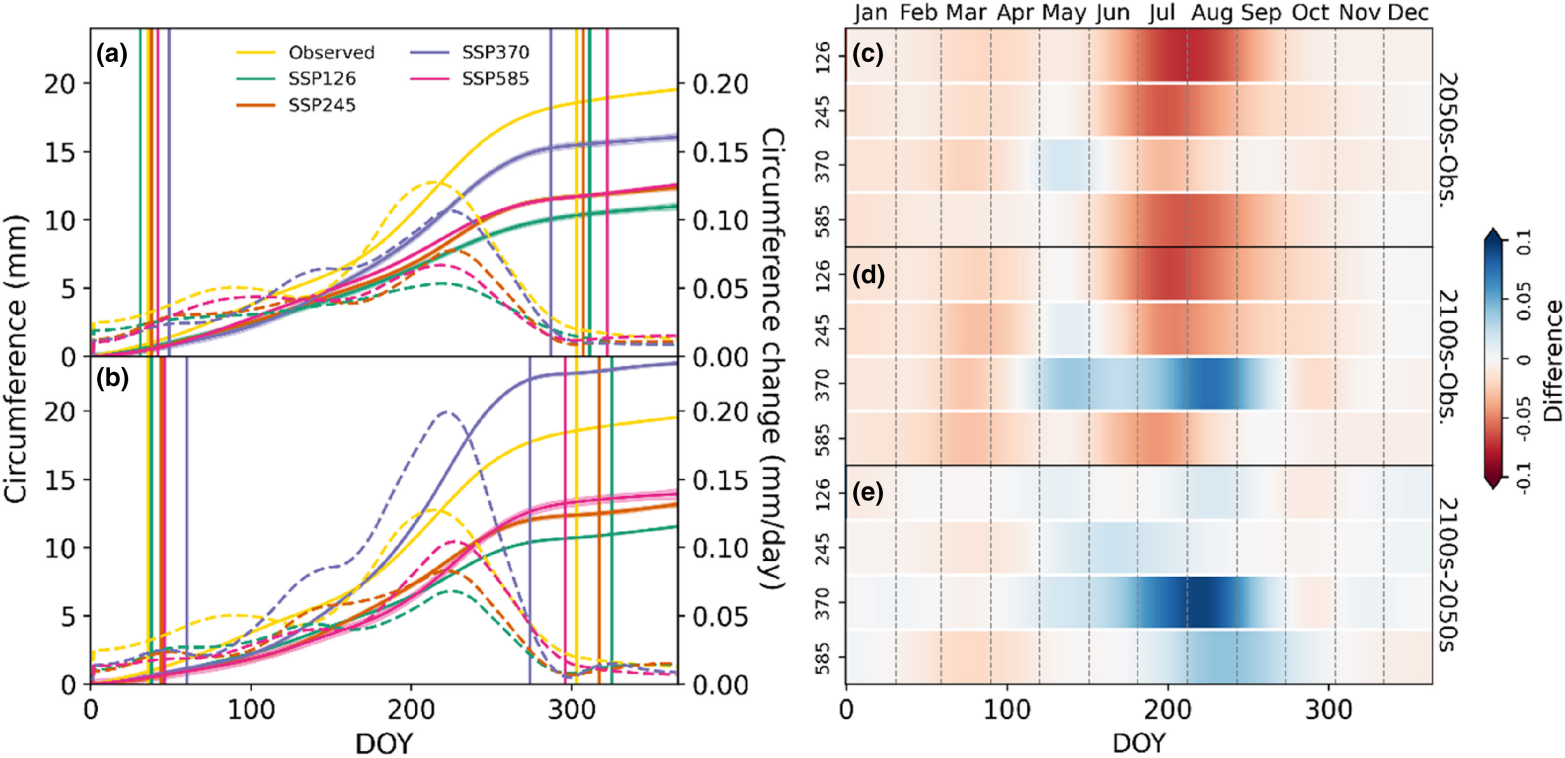
Annual circumferential growth patterns in the observed (Obs.) and modelled periods for different scenarios: (a) the 2050s and (b) the 2100s. The solid lines represent cumulative growth scaled in the left *y*‐axis, and the dashed lines represent the daily growth rate mounted in the right *y*‐axis. The vertical straight lines represent the beginning and cessation of the growing seasons, respectively. The *x*‐axis represents the day of the year (DOY). (c) and (d) represent the difference between the daily circumference change (DCC) rate in modelled and observation periods for the 2050s and 2100s, respectively, and (e) represents the difference between the DCC rate in the 2050s and 2100s.

In general, the total annual circumference growth of the investigated tree species will decrease in both analysed future periods (the 2050s and 2100s) and for all climate change scenarios (Figure 10). However, the intra‐annual growth pattern of the studied tree species will change as a result of changes in the distribution of DSCC clusters' occurrence. Like the cumulative growth, the daily growth rate of the trees will be lower than the observed growth rate, except in March for SSP375 during the 2050s, whereas during the 2100s for SSP275 and SSP370, trees will experience higher growth rates than during the observed period. In SSP370, trees will grow faster than in the observed period from the last week of April to the end of September. The statistically inferred start (SOS) and end (EOS) of the growing season of *P. roxburghii* using a 95% growth threshold were 36 and 303 in the observed period. On average, the statistically derived onset of the growing season will be delayed by 4 ± 7 and 11 ± 8 days in the 2050s and 2100s, respectively, irrespective of the SSP scenario. Similarly, the cessation of the growing season will be delayed by 4 ± 13 and 0 ± 20 days. The close examination of the growing season in each SSP revealed that the onset would start earlier only in SSP126, and trees will experience an early cessation of growth in SSP370 during the 2050s. On the contrary, all SSPs predict a later growth onset during the 2100s. However, SSP370 and SSP585 will experience an earlier cessation. Although the onset and cessation of the growing season are inconsistent between different scenarios, the overall growing season length will increase, except for SSP370 during both future periods and SSP585 during the 2100s.

## 4 DISCUSSION

The four DSCC clusters showed extreme intra‐annual seasonality (Figure 5). The seasonality of clusters demanded the inclusion of ‘months’ as one of the predictors in the classification model. This resulted in a distinct seasonality in the predictions (Figure 7). The comparison of the observed and predicted climatic conditions for cluster occurrence (Figure 6) indicated that the model performed consistently in all future periods using similar climate conditions for the prediction (Figure S4). However, their temporal distribution differed significantly due to changes in future temperature and precipitation seasonality. This consistency of cluster classification in present and future periods highlights the major role of environmental variables in defining distinct daily growth patterns. The precipitation and temperature conditions are important in defining the daily growth pattern by controlling the soil moisture and evapotranspiration (Deslauriers et al., 2003; Vospernik et al., 2020). The INM‐CM5‐0 CM projections indicate increased temperatures during the dry seasons (pre‐monsoon, post‐monsoon and winter) and enhanced precipitation amounts except for the summer monsoon season (Figure S5). This change in climate conditions results in a drier monsoon season but warmer and moister post‐monsoon and early‐winter seasons. The intra‐annual weather pattern is explained more clearly by the MI. The average annual MI is expected to decrease in future scenarios, primarily due to higher temperatures (Table S2). The change in intra‐annual weather patterns explains the future distribution of DSCC clusters and SCV changes of the trees.

The increased cluster0 during the pre‐monsoon and monsoon seasons is due to temperature depression during the monsoon season in the 2050s and 210s (Figure 7; Figures S6 and S7). Due to its frequent occurrence during cooler and drier conditions in the observation period, we assume its future increase is caused by the predicted cooler temperatures accompanied by less precipitation during July and August. The other non‐growing cluster2 is predicted to occur more often during the pre‐monsoon season, with a reduced occurrence during the post‐monsoon season. This result reflects the warmer and drier conditions in the pre‐monsoon period and relatively wetter conditions in the post‐monsoon, resulting in a higher occurrence of the GCs cluster1 and cluster3. Due to the optimum moisture availability in the summer monsoon season (Figure 7; Figures S8 and S9), the two GCs were predicted to be more concentrated within the growing season. The small peak of cluster1 during March–April in the 2050s might be due to an expected increase in precipitation. Its reduction in March–April during the 2100s can be attributed to drier conditions caused by increased PET. High PET at the beginning of the growing season may result in drier soil conditions, hampering plant growth (Körner, 2015). The balance between precipitation and PET (i.e. MI) is a proxy for soil water availability, directly influencing plant growth by modulating the stomatal conductance (Eckes‐Shephard et al., 2021). The warmer and wetter climate will encourage the occurrence of cluster3, which occurs during the post‐monsoon periods. Due to the decrease in July and August temperatures and sufficient summer monsoon precipitation, the occurrence of cluster1 and cluster3 will significantly increase during the 2100s, especially for SSP370 and SSP585. The variability in cluster occurrences across seasons offers insight into the adaptability and resilience of the tree species under changing climatic conditions. It suggests that trees will exhibit fluctuating growth responses even to the modest seasonal shifts in climate. These shifts can eventually influence the habitat suitability of species, impacting carbon sequestration (Case et al., 2021).

The average cumulative circumference is expected to reduce in the 2050s in all scenarios, whereas, in the 2100s, the trees will grow 20.4% more in the SSP370 scenario compared with the observed growth. In the 2050s, under SSP585, the warming in autumn and winter will shrink the length of the low‐temperature period, providing an opportunity for trees to grow in autumn and even in early winter (December). The warmer and wetter conditions during autumn and early winter might instead result in a higher occurrence of GCs. Even though pine trees may start growing in late winter, around DOY 40 in mid‐February, the dry pre‐monsoon period will likely hamper their growth temporarily. A decline in daily growth rate around this time of the year depicts this growth pause (Figure 10a,b). Such growth pauses might result in intra‐annual density fluctuations (IADFs). The frequent occurrence of IADFs might indicate that the tree species is highly responsive or vulnerable to environmental fluctuations (Battipaglia et al., 2023). With frequent occurrences of IADFs, the species might be at higher risk in changing climates compared with species with more consistent growth patterns.

The trees will likely experience decreased growth rates during the 2100s due to more pronounced dry pre‐monsoon conditions. A study on Himalayan cedar (*Cedrus deodara* G.Don) predicted a similar annual growth reduction for future climate conditions due to increasing drought intensity and frequency (Bhattacharyya et al., 2023). Warming‐induced high evapotranspiration reduces soil water content, photosynthesis, and carbohydrate reserves, simultaneously increasing the atmospheric demand for water vapour. Therefore, drought eventually deteriorates the water status in leaf and stem tissues, limiting tree growth (Wang et al., 2019). In contrast, the growth rate will increase in the summer monsoon season and even top the observed value in SSP370 during the 2100s, despite the decline in expected precipitation amount. It is important to note that trees need a critical amount of moisture or precipitation to start and maintain growth, which varies with geographical location. For instance, in the semi‐arid Tibetan Plateau, the necessary amount of precipitation was reported to be 17 mm (Ren et al., 2018). In our study site, the MI is positive in the monsoon season in both future periods, meaning precipitation exceeds PET. Therefore, enhanced growth during the summer monsoon will likely be controlled by temperatures instead of rainfall, as evidenced by other studies (eg., Aryal et al., 2023; Aryal, Gaire, et al., 2020). As discussed above, cool and moist weather conditions encourage the occurrence of cluster1, significantly increasing the growth rate, especially in July and August. The faster growth rate of trees under SSP370 during the 2100s combined with a shorter growing season can lead to the formation of wood with reduced wood density (Pretzsch et al., 2018) and an earlier dye‐off, consequently reducing the carbon storage capacity of the forest ecosystem (Brienen et al., 2020). Hence, the expected alterations in growth patterns, controlled by both temperature and precipitation, signal a potential shift in forest dynamics, affecting timber yields and influencing carbon uptake rates. Future forest management practices may need to focus on supporting species like *P. roxburghii* that might be at risk due to the climatic shifts.

The growing season in both future periods will be delayed and extended under most SSPs. However, in addition to SSP370 during the 2050s and 2100s, SSP585 will also experience a shrinking growing season during the 2100s. The extension of growing season length might have several positive and negative implications for trees. In boreal forests, longer growing seasons are expected to increase forest productivity due to enhanced carbon sequestration (Devi et al., 2020; Grossiord et al., 2022). However, the growing season extension also means more favourable conditions for the proliferation of pests, potentially leading to outbreaks and increased damage to tree populations. In addition, the future climate might not be favourable for every tree species, which might increase the dominance of one species, reducing biodiversity, which ultimately can hamper the resilience and resistance of the forest (Morin et al., 2018). The predicted delay in the growing period of *P. roxburghii* in subtropical climate can be linked with expected drier conditions (negative MI), especially during March–April, as evidenced by previous results derived from tree‐ring width chronologies (Aryal et al., 2018; Sigdel et al., 2018). A regional study based on remote‐sensing data in the Hindu Kush Himalaya predicted a similar increase in the growing season length of subtropical pine species (Shrestha et al., 2019). However, the observation‐based identification of the growing season of *P. roxburghii* from the study site is still lacking.

As a data‐driven statistical model, INTRAGRO predicts radial tree growth based on observations, using sub‐daily dendrometer data and daily climate data as inputs. It learns the complex growth–climate relationships from observed data and transfers the same relationships to future climate conditions using similar sets of input data. During the training of the classification model, INTRAGRO provides flexibility for users to incorporate additional environmental variables, which can affect a tree's intra‐annual growth. Adding more predictor variables gives it a broader perspective of the underlying patterns and relationships within the input environmental data. As a result, it becomes better trained to capture complex dependencies and make more accurate predictions, providing a better understanding of the factors influencing the outcome variable (Zhang et al., 2000). Dendrometers have evidently been used to study intra‐annual growth variation of a wide range of tree species from various environmental conditions. On the contrary, CMs also provide predictions on a global scale. Hence, INTRAGRO can be applied globally, regardless of tree species, climate and geographical conditions. INTRAGRO is based on machine learning algorithms for clustering and classification. These algorithms produce more robust results for large datasets. Hence, using only a short dendrometer series, which may not capture all possible growth responses under rare extreme climatic conditions, might limit the robustness of model results. However, the amount of data needed for model training also depends on how sensitive the predictant variable (in our case, ‘trees' circumference’) responds to the predictor variables (in our case ‘observed climate’) (Althnian et al., 2021). On the contrary, shorter dendrometer series, which are of limited scientific value, may also improve the model calibration and are, therefore, still useful. Including additional environmental variables, such as relative humidity, soil moisture, and drought indices, may further enhance the growth model's predictive power.

Furthermore, the dendrometer data may not directly represent the cambial activities because it might also record the bark growth, which is still an unsolved concern in the dendrometer community. However, the primary objective of INTRAGRO is to predict intra‐annual growth dynamics. The predicted growth can be further processed using existing approaches (e.g. Fan et al., 2019; McMahon & Parker, 2015; Miller et al., 2022) to infer the critical dates. The dependency of INTRAGRO on CM output makes it sensitive to the reliability of the used CM. Furthermore, INTRAGRO does not include factors like pests, diseases, human activities or unexpected environmental events (e.g. wildfires), which might also affect tree growth.

## 5 CONCLUSIONS

Our INTRAGRO approach performed well in the example dataset, yielding a robust prediction of future tree growth under different climate change scenarios. The approach underlined its potential use in modelling the intra‐annual growth pattern of tree species under different climate conditions, as revealed by a bundle of future climate scenarios.

INTRAGRO outlined the expected shifts in cluster occurrences across seasons, effectively revealing the plasticity of tree species in response to climatic variations. This adaptation emphasises the potential consequences of even subtle changes in intra‐annual climate on habitat suitability and carbon sequestration mechanisms. The projected fluctuations in cumulative circumference and growth rates point to the vulnerability of forests in the future. These observed patterns are driven by the coupled effect of temperature and moisture availability.

The output of INTRAGRO significantly advances our understanding of tree growth in subtropical regions, shedding light on the complex dynamics of climatic changes and their subsequent impacts on forest ecosystems. Such insights are invaluable, as they offer a view into the future health and viability of these critical ecosystems and guide conservation and forest management practices to mitigate the impact of rapid climate change. This study demonstrates the applicability of INTRAGRO using a species growing in a humid subtropical area. Given the increasingly wide applicability of dendrometers and global coverage of CMs generating robust predictions, INTRAGRO allows us to expand our understanding of future tree growth on a global scale. We move forward by testing the approach in other climate zones, for example temperate/boreal climate, dry and humid tropics, and continued research in this domain and adaptive strategies will be essential to safeguarding our forest ecosystems.

## AUTHOR CONTRIBUTIONS

**Sugam Aryal:** Conceptualization (equal); data curation (lead); formal analysis (lead); investigation (lead); methodology (lead); software (lead); validation (equal); visualization (lead); writing – original draft (lead); writing – review and editing (equal). **Jussi Grießinger:** Formal analysis (equal); funding acquisition (equal); supervision (supporting); writing – original draft (equal); writing – review and editing (equal). **Nita Dyola:** Methodology (supporting); writing – original draft (supporting); writing – review and editing (supporting). **Narayan Prasad Gaire:** Resources (supporting); writing – review and editing (supporting). **Tribikram Bhattarai:** Resources (supporting); writing – review and editing (supporting). **Achim Bräuning:** Conceptualization (lead); methodology (supporting); project administration (lead); resources (lead); supervision (lead); writing – original draft (equal); writing – review and editing (lead).

## Supporting information


Data S1

## Data Availability

All the dataset and codes will be made available at https://doi.org/10.5061/dryad.mw6m90633 and https://github.com/sugam72‐os/INTRAGRO.git.
