# Supplementary material for: INTRAGRO: A machine learning approach to predict future growth of trees under climate change
Source: Ecol Evol. 2023 Oct 20;13(10):e10626. doi: 10.1002/ece3.10626 (PMC10587741; doi:10.1002/ece3.10626)
Supplement: Supplementary file 1 — Data S1 [file ECE3-13-e10626-s001.docx]

INTRAGRO: A machine learning approach to predict future growth of terrestrial ecosystems under climate change

Sugam Aryal^1^*, Jussi Grießinger^1^, Nita Dyola^2,3^, Narayan Prasad Gaire^4^, Tribikram Bhattarai^5^, Achim Bräuning^1^

^1^Friedrich-Alexander-Universität Erlangen-Nürnberg, Institut für Geographie
Wetterkreuz 15, Erlangen, Bayern, DE 91058

^2^Institute of Tibetan Plateau Research, Chinese Academy of Sciences, State Key Laboratory of Tibetan Plateau Earth System, Resources and Environment (TPESRE), Beijing, CN 100101

^3^Laboratoire sur les écosystèmes terrestres boréaux, Département des Sciences Fondamentales, Universitédu Québec à Chicoutimi, Chicoutimi G7H2B1, Canada

^4^Department of Environmental Science, Patan Multiple Campus, Tribhuvan University, Lalitpur, 44700, Nepal

^5^Central Department of Biotechnology, Tribhuvan University, Kathmandu, 44600, Nepal

*Author for correspondence: Sugam Aryal (sugam.aryal@fau.de)

Supporting information


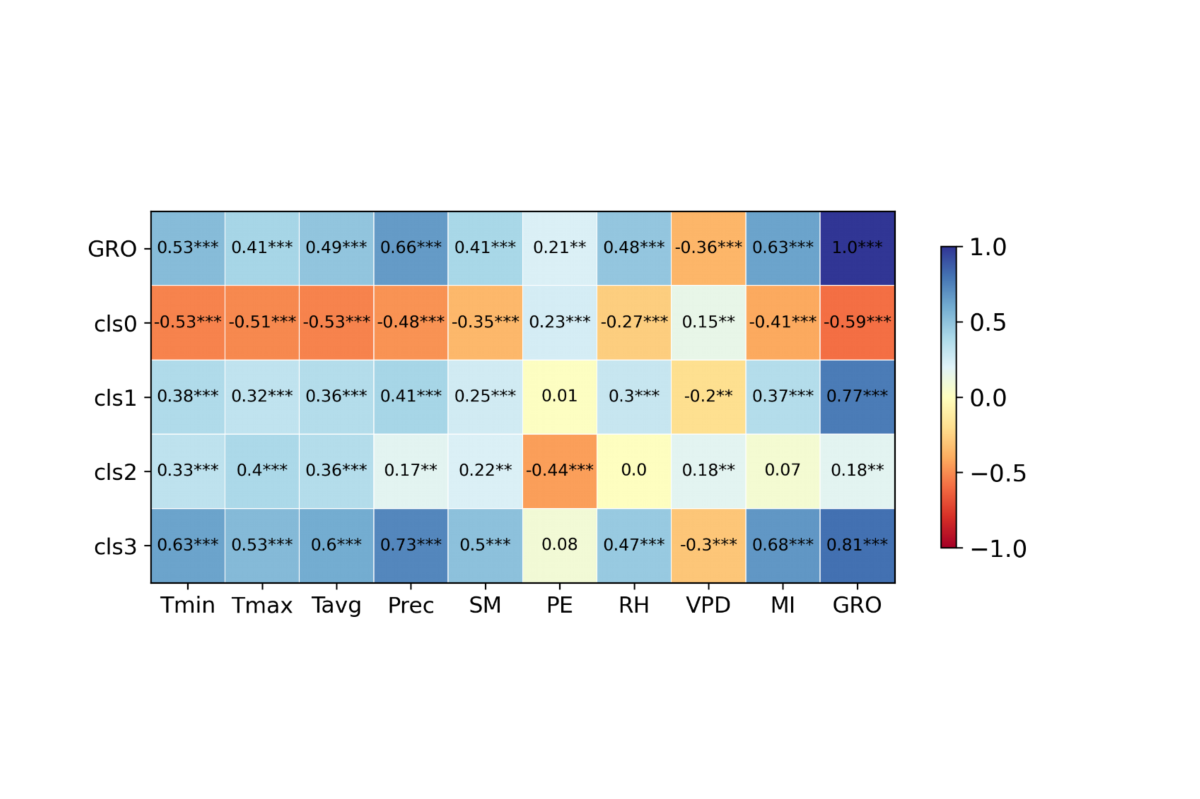


*Figure S 1. Correlations of monthly aggregated cluster numbers with climate variables. cls* represents cluster class and ‘Tmin’, ‘Tmax’, ‘Tavg’, ‘Prec’, ‘SM’, ‘PE’, ‘RH’, ‘VPD’,* ‘MI*’, and ‘GRO’ represent minimum temperature, maximum temperature, average temperature, precipitation, soil moisture, potential evaporation, relative humidity, vepour pressure deficit, moisture index and monthly growth, respectively.*


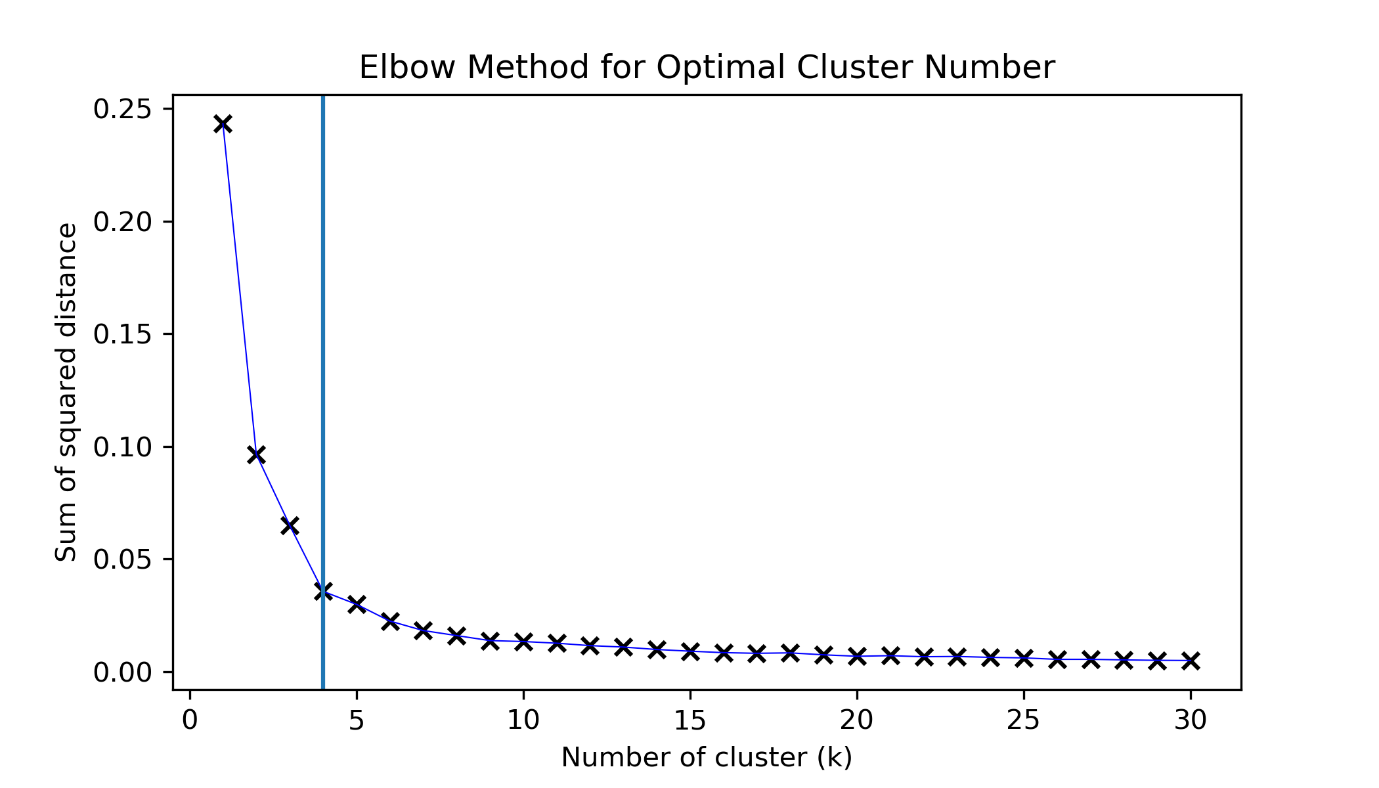


*Figure S 2. Identification of the optimum number of clusters using the Elbow method.*


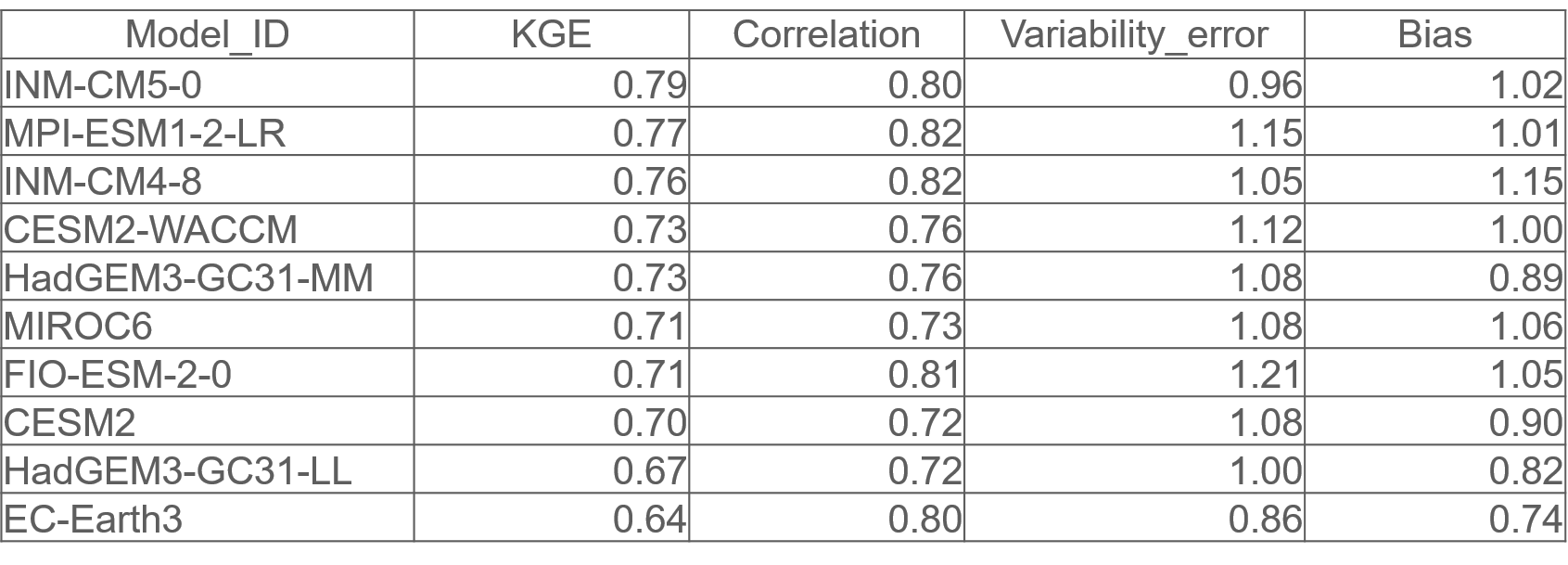

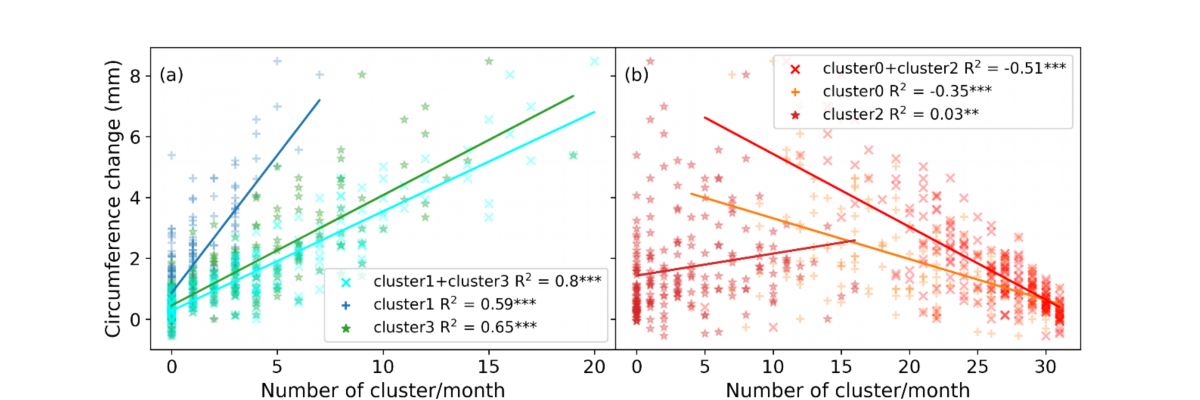


*Figure S 3. Regression analysis between monthly aggregated cluster number and stem circumference change. ‘cluster0+cluster2’ represents the combination of cluster0 and cluster2, and ‘cluster1+cluster3’ represents the combination of cluster1 and cluster3.*


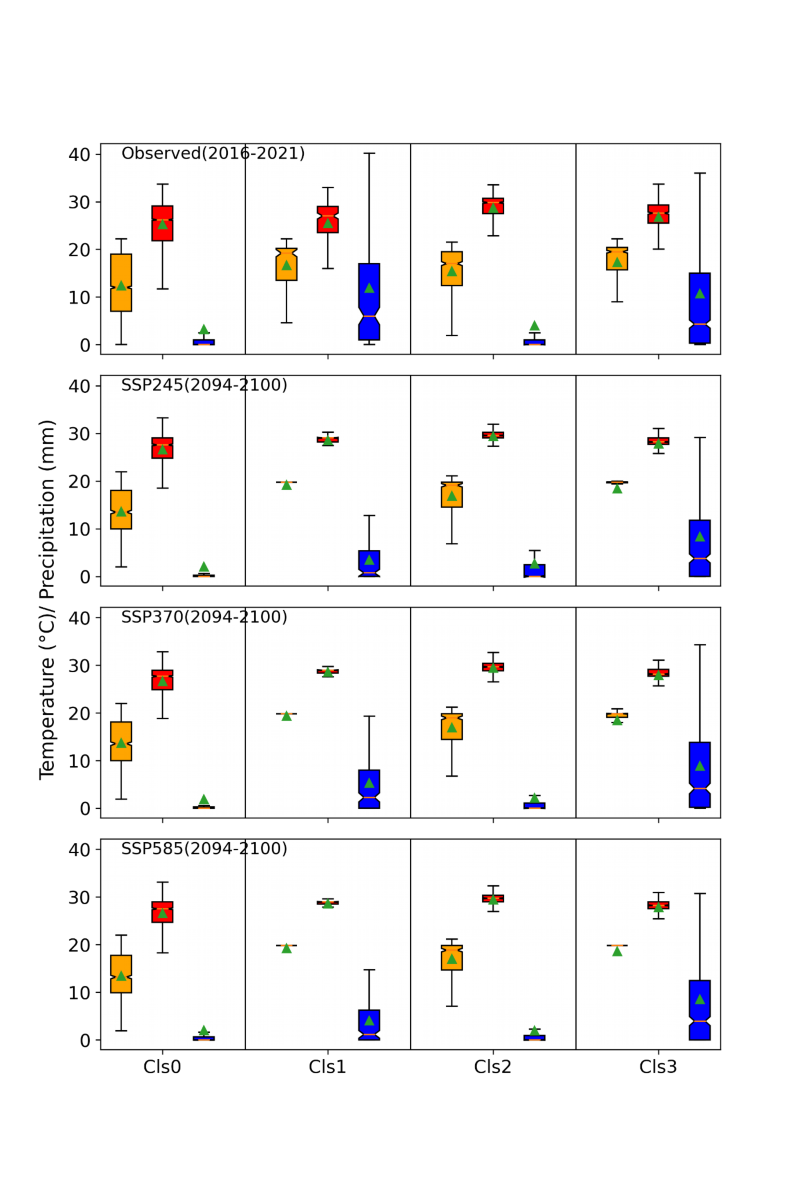


*Figure S 4. Comparison of climate conditioned for the occurrence of each cluster class in the observation period and for the end of the 21^st^ century for different climate change scenarios.*


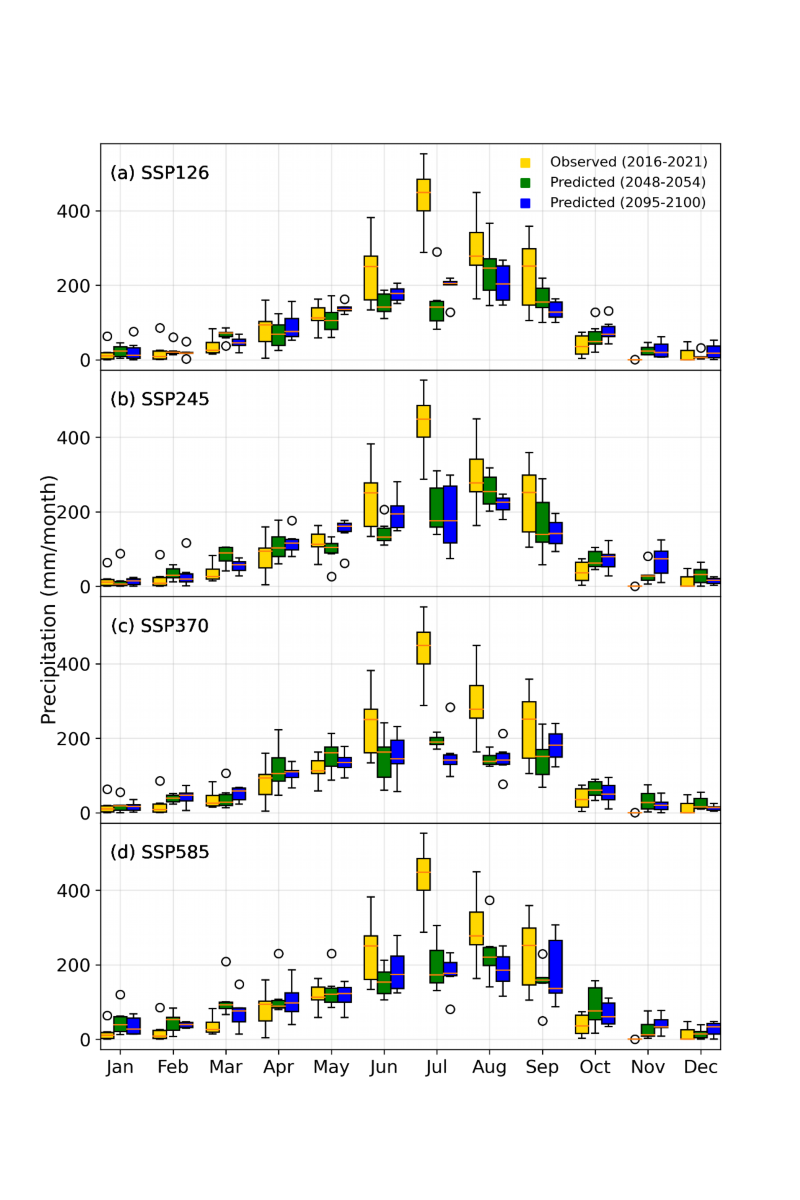


*Figure S* 5*. Comparison of monthly precipitation amounts in the observation period and for different modeled periods under climate change scenarios.*


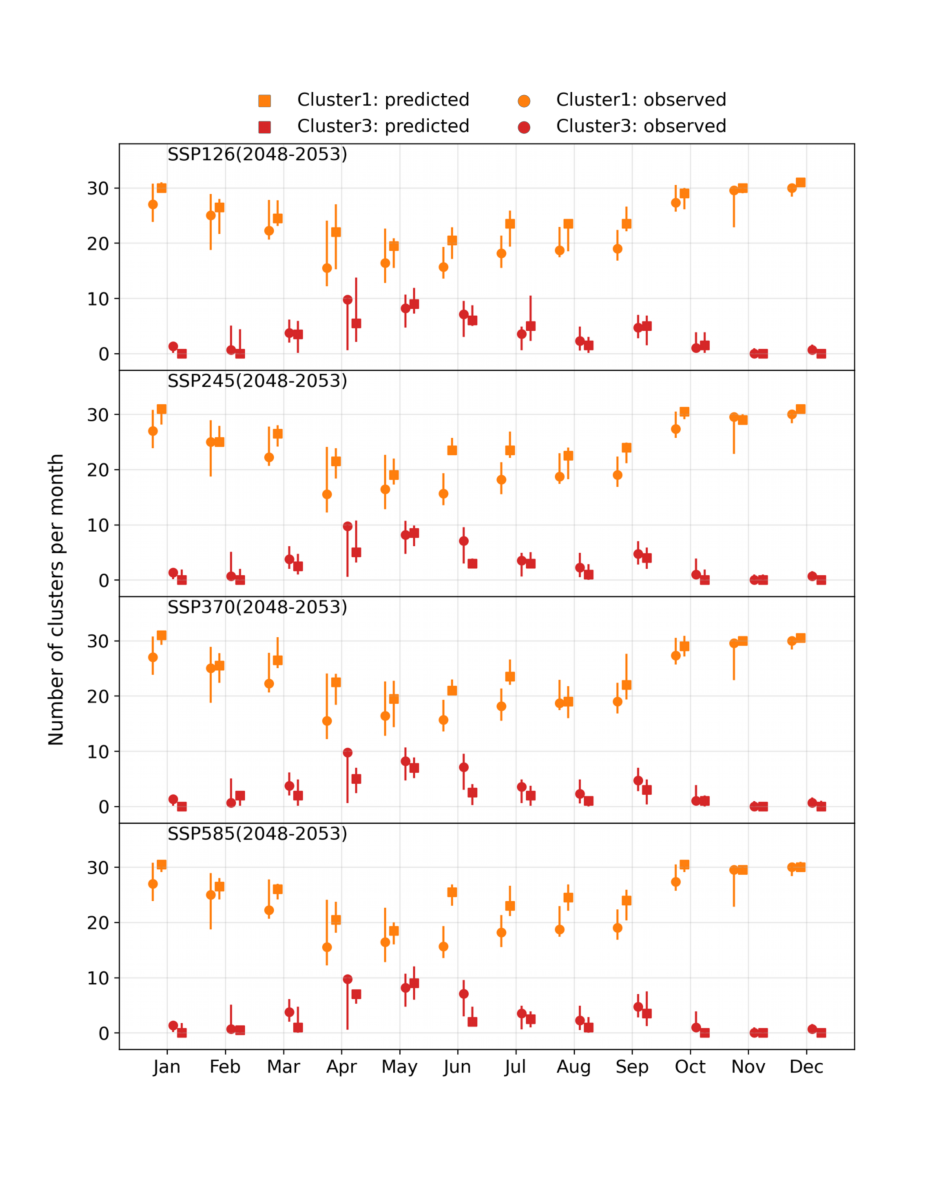


*Figure S 6. Occurrence of cluster0 and cluster2 in the observation period and for the middle of the 21^st^ century for different climate change scenarios.*


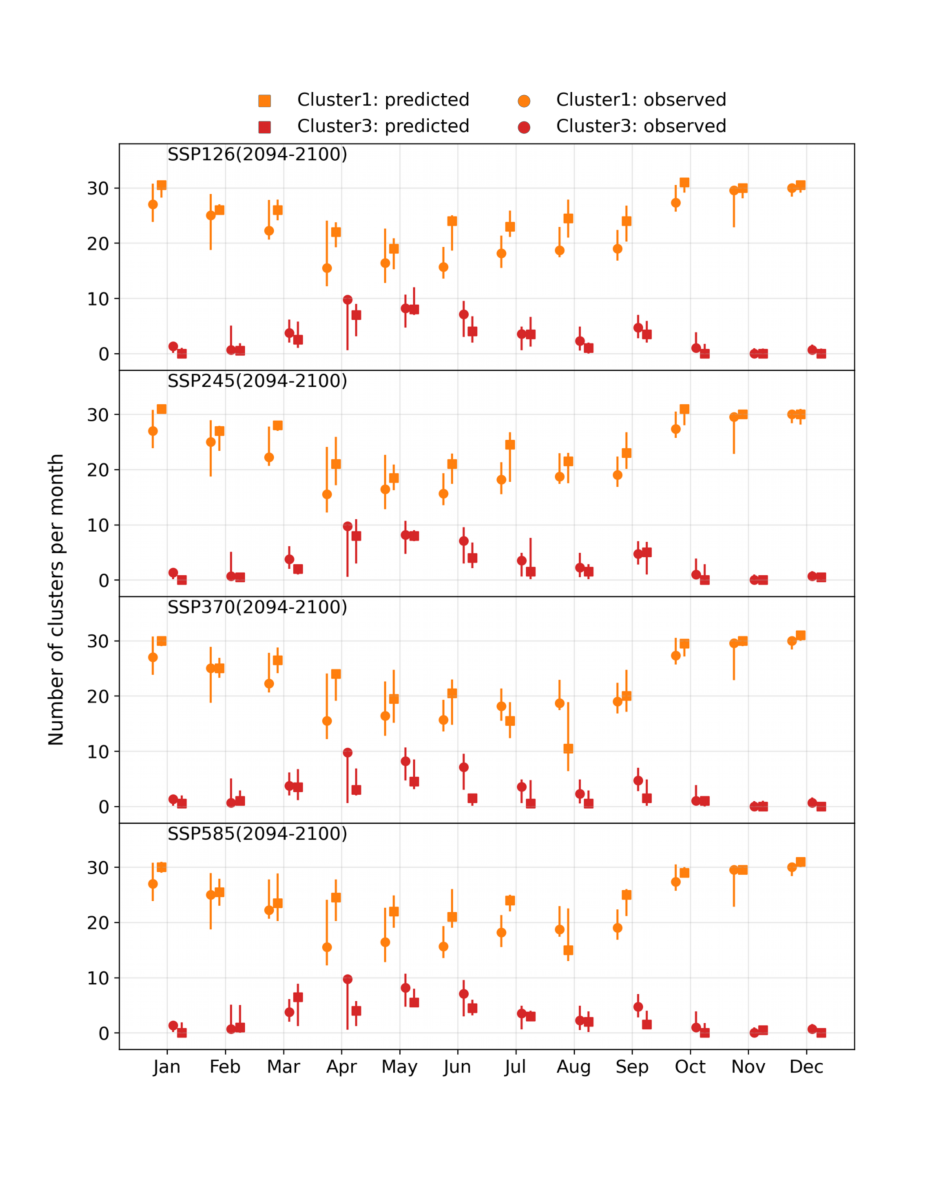


*Figure S 7. Occurrence of cluster0 and cluster2 in the observation period and for the end of the 21^st^ century for different climate change scenarios.*


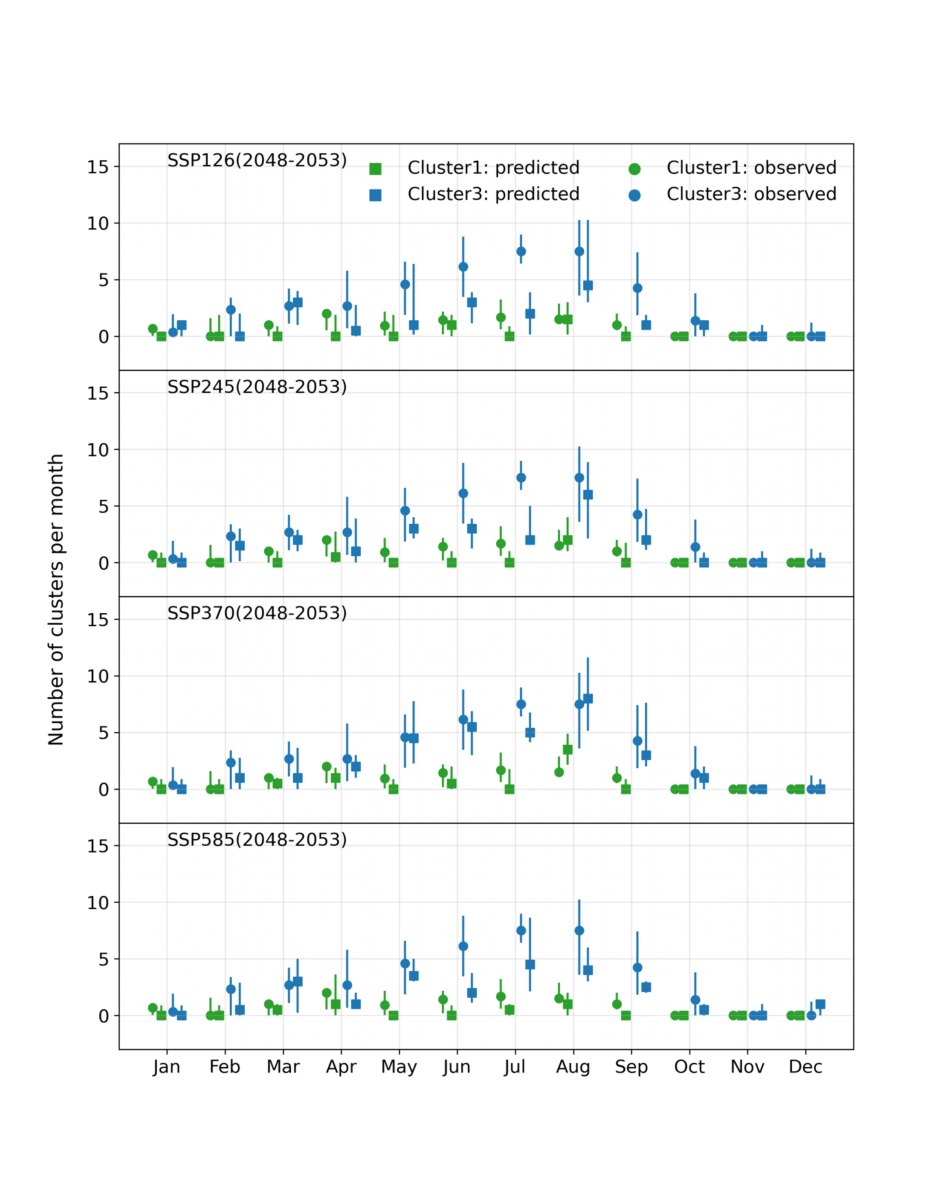


*Figure S 8. Occurrence of cluster1 and cluster3 in the observation period and for mid of the 21^st^ century for different climate change scenarios*


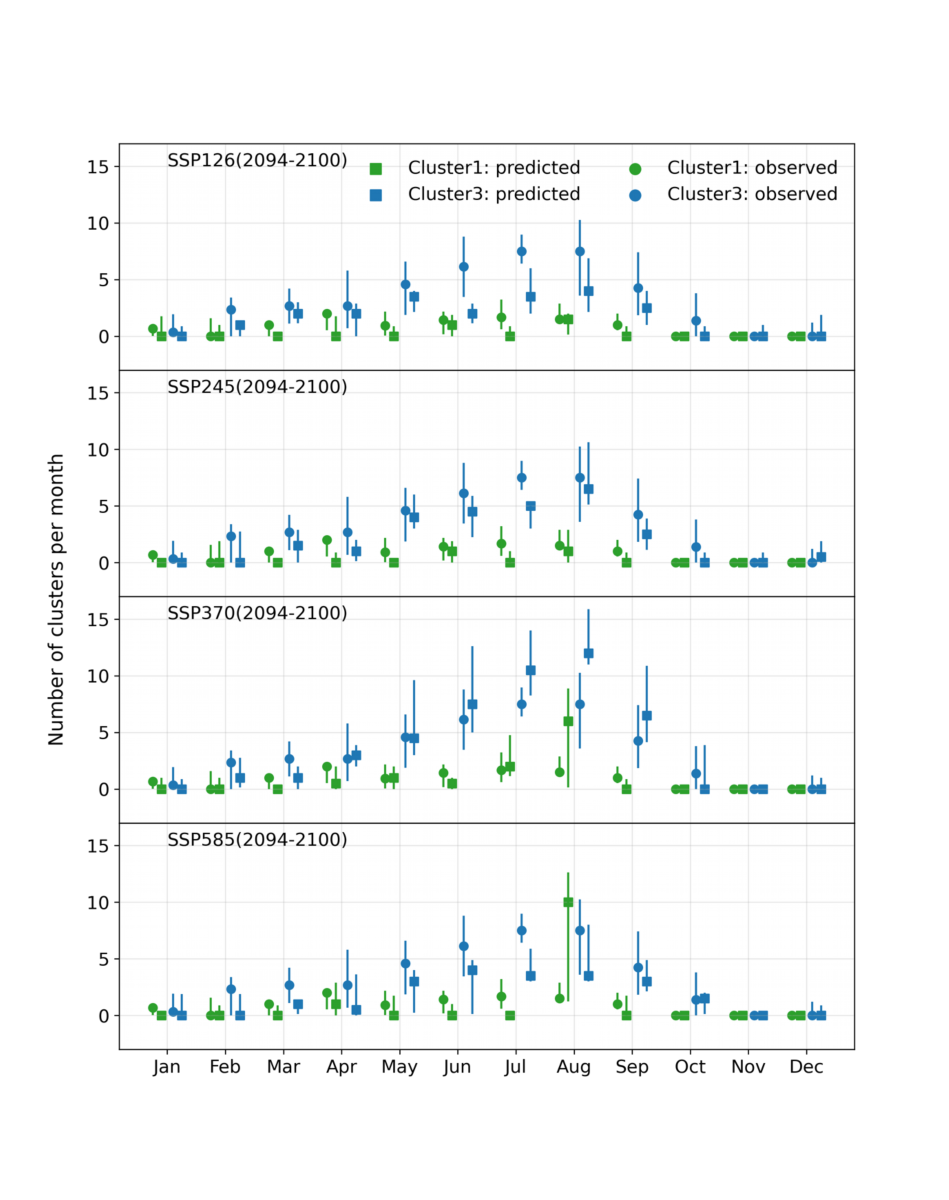


*Figure S 9. Occurrence of cluster1 and cluster3 in the observation period and for mid of the 21^st^ century for different climate change scenarios*


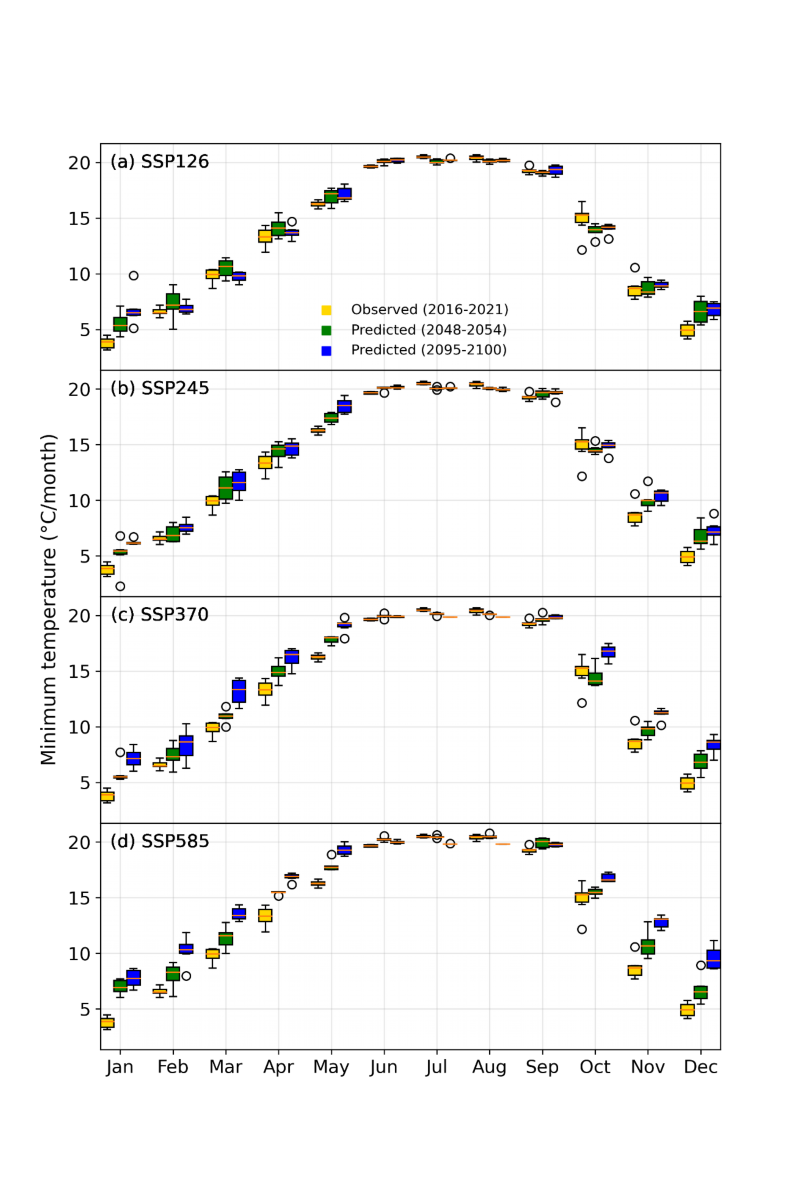


*Figure S 10. Comparison of monthly minimum temperatures in the observation period and for different modeled periods under climate change scenarios.*


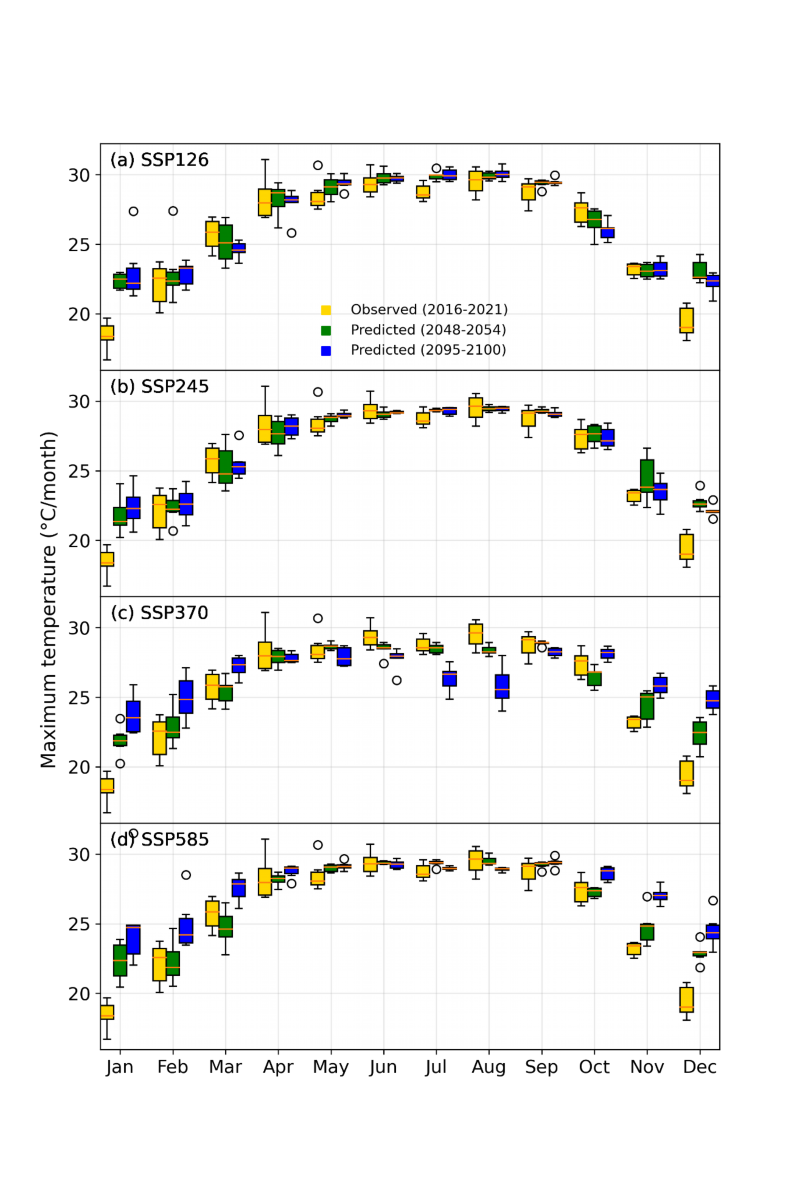


*Figure S 11. Comparison of monthly maximum temperatures in the observation period and for different modeled periods under climate change scenarios.*

*Table S 1. List of the 10 best fitting climate models for minimum and maximum temperature and precipitation.*

| **Parameter** | **Model_ID** | **KGE** | **Correlation** | **Variability_error** | **Bias** |
| --- | --- | --- | --- | --- | --- |
| Maximum Temperature | INM-CM4-8 | 0.88 | 0.97 | 1.12 | 1.02 |
|  | MIROC-ES2L | 0.87 | 0.95 | 0.88 | 0.96 |
|  | INM-CM5-0 | 0.80 | 0.97 | 1.19 | 0.97 |
|  | CIESM | 0.73 | 0.97 | 1.17 | 1.20 |
|  | MPI-ESM1-2-LR | 0.69 | 0.94 | 1.01 | 1.31 |
|  | IPSL-CM6A-LR | 0.65 | 0.98 | 1.21 | 1.28 |
|  | CNRM-CM6-1-HR | 0.62 | 0.95 | 1.20 | 1.32 |
|  | CNRM-CM6-1 | 0.62 | 0.93 | 0.99 | 1.38 |
|  | HadGEM3-GC31-LL | 0.61 | 0.97 | 0.98 | 1.39 |
|  | BCC-CSM2-MR | 0.60 | 0.93 | 0.90 | 1.38 |
| Minimum Temperature | INM-CM4-8 | 0.73 | 0.83 | 1.20 | 0.92 |
|  | INM-CM5-0 | 0.67 | 0.83 | 1.27 | 0.94 |
|  | MIROC-ES2L | 0.53 | 0.85 | 1.42 | 0.84 |
|  | IPSL-CM6A-LR | 0.48 | 0.86 | 1.49 | 1.12 |
|  | MPI-ESM1-2-LR | 0.45 | 0.83 | 1.52 | 1.00 |
|  | EC-Earth3 | 0.44 | 0.79 | 1.48 | 1.19 |
|  | GISS-E2-1-G | 0.42 | 0.76 | 1.25 | 1.46 |
|  | EC-Earth3-Veg | 0.39 | 0.79 | 1.54 | 1.19 |
|  | BCC-CSM2-MR | 0.34 | 0.86 | 1.64 | 1.10 |
|  | NESM3 | 0.30 | 0.90 | 1.67 | 0.83 |
| Precipitation | INM-CM5-0 | 0.79 | 0.80 | 0.96 | 1.02 |
|  | MPI-ESM1-2-LR | 0.77 | 0.82 | 1.15 | 1.01 |
|  | INM-CM4-8 | 0.76 | 0.82 | 1.05 | 1.15 |
|  | CESM2-WACCM | 0.73 | 0.76 | 1.12 | 1.00 |
|  | HadGEM3-GC31-MM | 0.73 | 0.76 | 1.08 | 0.89 |
|  | MIROC6 | 0.71 | 0.73 | 1.08 | 1.06 |
|  | FIO-ESM-2-0 | 0.71 | 0.81 | 1.21 | 1.05 |
|  | CESM2 | 0.70 | 0.72 | 1.08 | 0.90 |
|  | HadGEM3-GC31-LL | 0.67 | 0.72 | 1.00 | 0.82 |
|  | EC-Earth3 | 0.64 | 0.80 | 0.86 | 0.74 |

Table S 2: Change in climate parameters in two future periods: 2050s and 2100s with respect to the corresponding climate during the observation period. The precipitation and moisture index change is the percentage change, whereas minimum and maximum temperatures are expressed as ^o^C. The cells filled with red and green colors represent negative and positive change, respectively.

| Climate | Period | Scenario | Jan | Feb | Mar | Apr | May | Jun | Jul | Aug | Sep | Oct | Nov | Dec | Annual |
| --- | --- | --- | --- | --- | --- | --- | --- | --- | --- | --- | --- | --- | --- | --- | --- |
| Precipitation | 2050s | SSP126 | 10.08 | 21.67 | 62.91 | -23.90 | -5.86 | -37.69 | -65.32 | -18.39 | -30.49 | 60.05 | more than 100 | 47.17 | -28.98 |
|  |  | SSP245 | -7.94 | 61.14 | 102.96 | 20.57 | -17.35 | -39.79 | -52.49 | -13.12 | -29.05 | 85.49 | more than 100 | 348.23 | -19.02 |
|  |  | SSP370 | -8.03 | 83.47 | 1.37 | 31.98 | 32.54 | -38.63 | -55.95 | -51.47 | -37.35 | 64.08 | more than 100 | 281.81 | -27.14 |
|  |  | SSP585 | 132.49 | 119.57 | 162.11 | 24.32 | 14.87 | -35.51 | -54.69 | -21.14 | -34.52 | 129.42 | more than 100 | 129.00 | -14.60 |
|  | 2100s | SSP126 | 11.85 | -1.28 | 11.08 | -1.04 | 19.72 | -26.08 | -55.42 | -30.46 | -43.12 | 103.02 | more than 100 | 229.21 | -25.03 |
|  |  | SSP245 | -40.31 | 62.32 | 31.94 | 29.72 | 26.74 | -17.35 | -57.29 | -25.97 | -38.36 | 90.44 | more than 100 | 118.72 | -17.71 |
|  |  | SSP370 | -22.89 | 103.10 | 23.97 | 15.16 | 17.69 | -36.17 | -63.80 | -51.40 | -21.97 | 36.76 | more than 100 | 92.83 | -30.33 |
|  |  | SSP585 | 70.37 | 85.76 | 78.43 | 14.06 | 0.80 | -22.35 | -59.82 | -37.26 | -21.37 | 77.56 | more than 100 | 318.42 | -19.87 |
| Moisture Index (MI) | 2050s | SSP126 | -24.39 | 1.97 | 32.11 | -66.11 | -28.00 | -104.09 | -101.68 | -38.98 | -68.99 | 33.25 | 28.06 | -12.80 | -413.57 |
|  |  | SSP245 | -32.93 | 17.84 | 51.20 | 25.07 | -46.05 | -101.93 | -80.93 | -26.56 | -63.53 | 41.00 | 30.60 | 20.97 | -269.40 |
|  |  | SSP370 | -31.67 | 22.61 | 3.91 | 47.84 | 91.86 | -92.60 | -82.92 | -92.82 | -77.60 | 38.99 | 32.01 | 16.43 | -338.76 |
|  |  | SSP585 | 26.41 | 38.97 | 82.49 | 32.18 | 38.34 | -92.45 | -83.27 | -40.45 | -72.79 | 70.31 | 24.96 | -2.97 | -201.37 |
|  | 2100s | SSP126 | -24.80 | -4.95 | 10.64 | -22.68 | 35.33 | -70.98 | -87.07 | -62.94 | -95.59 | 60.59 | 29.85 | 11.60 | -358.63 |
|  |  | SSP245 | -43.98 | 18.29 | 19.56 | 36.35 | 78.09 | -41.95 | -87.87 | -51.53 | -81.66 | 46.37 | 81.94 | 2.03 | -242.34 |
|  |  | SSP370 | -48.14 | 16.85 | 9.52 | 28.20 | 89.62 | -77.91 | -87.32 | -76.52 | -39.08 | 20.82 | 13.91 | -15.02 | -337.71 |
|  |  | SSP585 | -10.13 | 16.67 | 32.12 | 15.48 | 16.67 | -57.42 | -91.18 | -70.59 | -47.49 | 38.49 | 34.49 | 13.88 | -292.58 |
| Minimum temperature | 2050s | SSP126 | 1.92 | 0.66 | 0.80 | 1.13 | 0.64 | 0.43 | -0.43 | -0.30 | -0.19 | -0.97 | -0.15 | 1.77 | 0.44 |
|  |  | SSP245 | 1.85 | 0.39 | 1.42 | 1.36 | 1.10 | 0.40 | -0.44 | -0.35 | 0.33 | -0.28 | 1.22 | 1.89 | 0.74 |
|  |  | SSP370 | 1.69 | 0.80 | 1.24 | 1.94 | 1.53 | 0.29 | -0.37 | -0.30 | 0.38 | -0.37 | 0.85 | 1.94 | 0.80 |
|  |  | SSP585 | 2.98 | 1.42 | 1.69 | 2.43 | 1.57 | 0.60 | -0.05 | 0.08 | 0.66 | 0.65 | 2.00 | 1.87 | 1.32 |
|  | 2100s | SSP126 | 2.62 | 0.30 | 0.02 | 0.72 | 0.85 | 0.55 | -0.28 | -0.22 | 0.03 | -0.79 | 0.19 | 1.93 | 0.49 |
|  |  | SSP245 | 2.40 | 0.99 | 1.88 | 1.67 | 2.21 | 0.50 | -0.42 | -0.47 | 0.34 | -0.01 | 1.58 | 2.42 | 1.09 |
|  |  | SSP370 | 3.24 | 1.76 | 3.47 | 3.19 | 2.80 | 0.25 | -0.65 | -0.56 | 0.58 | 1.84 | 2.34 | 3.50 | 1.81 |
|  |  | SSP585 | 3.88 | 3.61 | 3.84 | 3.82 | 3.02 | 0.35 | -0.69 | -0.60 | 0.48 | 1.94 | 4.00 | 4.72 | 2.36 |
| Maximum temperature | 2050s | SSP126 | 3.71 | 1.14 | -0.33 | 0.54 | 0.64 | 0.44 | 1.17 | 0.38 | 0.58 | -0.81 | -0.21 | 3.51 | 0.90 |
|  |  | SSP245 | 3.80 | 0.45 | -0.20 | -0.06 | 0.27 | -0.30 | 0.61 | -0.05 | 0.54 | 0.18 | 1.10 | 3.20 | 0.80 |
|  |  | SSP370 | 3.55 | 1.02 | -0.02 | 0.17 | 0.19 | -0.91 | -0.25 | -1.14 | 0.10 | -0.94 | 1.13 | 2.79 | 0.47 |
|  |  | SSP585 | 4.13 | 0.36 | -0.76 | 0.49 | 0.54 | 0.00 | 0.61 | 0.00 | 0.44 | -0.16 | 1.45 | 3.35 | 0.87 |
|  | 2100s | SSP126 | 4.12 | 1.02 | -0.85 | 0.22 | 0.93 | 0.36 | 1.26 | 0.56 | 0.70 | -1.43 | -0.07 | 2.68 | 0.79 |
|  |  | SSP245 | 3.63 | 0.74 | 0.03 | 0.47 | 0.55 | -0.18 | 0.56 | -0.07 | 0.32 | -0.06 | 0.18 | 2.57 | 0.73 |
|  |  | SSP370 | 5.32 | 3.08 | 1.75 | 0.11 | -0.56 | -1.62 | -2.37 | -3.71 | -0.55 | 0.72 | 2.54 | 5.26 | 0.83 |
|  |  | SSP585 | 5.76 | 3.06 | 2.11 | 1.07 | 0.70 | -0.12 | 0.25 | -0.61 | 0.61 | 1.19 | 3.74 | 4.98 | 1.90 |
